# Effects of group-based physical activity programs on children, adolescents, and young adults with disabilities: A systematic review

**DOI:** 10.1371/journal.pone.0323707

**Published:** 2025-05-23

**Authors:** Jason D’Amours, Stéphanie Girard, Paule Miquelon, Pierre-Luc Veillette

**Affiliations:** 1 Department of psychology, Université du Québec à Trois-Rivières,; 2 Department of human kinetics, Université du Québec à Trois-Rivières,; 3 Department of psychology, Université du Québec à Trois-Rivières; Iran University of Medical Sciences, IRAN, ISLAMIC REPUBLIC OF

## Abstract

**Purpose:**

In recent years, a concerning decline in the physical activity levels of young individuals has been observed, with a notable impact on those with disabilities. Despite evidence suggesting that physical activity programs yield health benefits for youths with disabilities, there exists a significant gap in understanding the specific group-based effects, strategies for effectiveness, sustainability, program development, adaptation details, and empirical value.

**Method:**

This systematic review examines quasi-experimental and experimental design research published from 2013 to 2025 on group-based physical activity programs for children, adolescents, and young adults (5–24 years old) with disabilities according to the PRISMA guidelines.

**Results:**

The searches yielded 2 715 studies, of which 20 were included. This review highlights multiple disabilities, diverse program durations and activities, and favorable results spanning physical, social, emotional, and cognitive dimensions. The main findings indicated strategies for program effectiveness portrayed as adaptation details. Despite every study providing positive outcomes, limitation concerning confounding variables, sample size and disparity, generalizability, sustainability, and fidelity of implementation remain.

**Conclusions:**

Overall, the selected studies suggest great holistic outcomes for youth with disabilities and are to be implemented in addition to special schools or treatment. Future research should explore differences among less common disabilities, emphasizing cognitive outcomes, trainer impact, and program inclusivity to develop accessibility and effective interventions for youth with disabilities.

## Introduction

The physical activity (PA) levels of young people have decreased over the years [1]. Furthermore, youths (5–24 years old) with disabilities (e.g., autism spectrum disorder, specific learning disabilities, attention deficit disorder, etc.) seem even less physically active than those without disabilities [2]. Indeed, approximately 80% of children with disabilities do not respect the minimum of 60 minutes of PA per day of moderate to vigorous intensity [3,4]. Because physical inactivity is often linked to several chronic conditions later in life including obesity, heart disease, hypertension, stroke, colon and breast cancer, diabetes (type 2) and osteoporosis [5], promoting the PA participation of young people is essential.

PA is crucial to the social, affective, physical and cognitive development of children, adolescents and young adults with disabilities. Daily PA is characterized by improvements in several areas: social (relationships, communication), physical (motor skills, cardiometabolic health), cognitive (executive functions, attention) and affective (well-being, self-esteem) [6]. More specifically, it relates to greater endurance, strength, coordination, balance, weight loss, enhanced self-esteem, improved impulsivity management, anger and aggressivity control, concentration, mental flexibility, problem-solving skills and working memory [6,7]. Additional positive outcomes include a reduced risk of developing chronic diseases, stress reduction, increased energy levels, and enhanced social activity including peer interactions resulting in friendships [5]. It appears, then, that PA yields multiple benefits in several spheres of development for youths with disabilities.

Organized youth sports during childhood and adolescence may be a promising avenue for PA promotion as they are positively related to the frequency of leisure-time PA in adulthood [8–10]. However, one of the most common barriers to PA participation cited by parents is the lack of suitable programs for children with disabilities [11,12]. Indeed, programs and opportunities for PA participants are acknowledged to be limited compared to those with no disabilities [13]. The reason may be the many challenges of developing community-based programs for youths with disabilities, which include the lack of adaptive exercise equipment, the use of appropriate fitness assessments, transportation issues and the recruitment of qualified personnel [4–14]. Furthermore, youths with disabilities practice less PA because it is not adapted to their needs for various reasons (multiple disorders, complexity, minority status), which explains the scarcity of PA programs available for them [14,15]. Other concerns regarding those programs include access restrictions, lack of information about physical activities, lack of community support and the diversity of disabilities, which makes participation more difficult [16,17].

The lack of effective community and group-based programs for youths with disabilities limits opportunities for developmental improvement in the aforementioned areas [18]. Indeed, physical inactivity can affect basic body management skills such as stability and spatial awareness, making PA participation more difficult over time [19]. In addition, a lack of fundamental movement skills can lead to multiple issues such as insecurity and low self-esteem at an early age as children observe the difference between themselves and other children at school [20]. This early gap predisposes youths with disabilities to problems later in life including obesity, metabolic disorders, decrease in overall health condition, increase in sedentary behaviour and social isolation, all of which contribute to a lower quality of life [6–21].

As a result, individualized PA is often seen as an answer to these problems, since youths with disabilities are more likely to participate in solitary, sedentary and home-based leisure activities than in active PA or group-based and community leisure programs [22,23]. One advantage of individualized PA is the possibility of responding to an individual’s specific needs. A corresponding problem, however, is that individualized PA limits social opportunities and often includes therapy-based situations that require qualified individuals and resources. Indeed, although individual PA have some benefits for youths with disabilities, access to quality group-based programs represents an important aspect of their social development.

A previous study [24], for example, shows that PA programs help improve social communication skills in parallel with traditional interventions such as therapy or treatment and may reduce the severity of restricted and repetitive behaviours for children with autism spectrum disorder (ASD). Group-based PA is undoubtedly a rich social experience, as it creates a feeling of acceptance by peers and helps create opportunities for friendship [25]. Moreover, children and adolescents who participate in such programs often report they are motivated by their peers and have better social interactions [26]. Group activities also facilitate social and communication skills because team sports players support each other [27]. In general, participation in a group-based activity program can significantly help improve interpersonal and social skills [28]. All these benefits are important for young people with disabilities, as the social component is often clinically difficult to address, and group-based PA programs offer a way to correct this limitation. The social complexities faced by youths with disabilities underscore the clinical significance of the social component. Group-based PA programs stand as innovative solutions to address these issues, providing a nuanced approach to empower individuals with disabilities to navigate social interactions [27]. In the context of a systematic review, these insights establish the foundation for further exploration of the effects and implications of group-based PA programs for individuals with disabilities.

Although certain studies and reviews show that group-based PA is effective in interventions conducted with children, adolescents and young adults with disabilities, results regarding the effects remain unclear owing to the broad disparities in sample size, heterogeneity of interventions, intervention time, intervention frequency and measurement [14]. While there is evidence that PA programs provide some health benefits, little is known about specific group-based effects, strategies for effectiveness, sustainability, program development, adaptation details and the programs’ empirical value for youths with disabilities. The present systematic review, therefore, aims to identify the characteristics and effects of group-based PA programs for young people (5–24 years old) with disabilities.

## Method

This review is based on studies conducted over the last 12 years (2013–2025). The initial time frame focused on the last 10 years (2013–2023) to prioritize recent research on group-based PA programs for school-age children, adolescents, and young adults with disabilities. However, due to the extended completion period of the review, multiple updates were made to include newly published studies, which led to an expanded publication window. It is registered with the International Prospective Register of Systematic Reviews (PROSPERO) (CRD42023392682) and adheres to the guidelines in the PRISMA Statement (Preferred Reporting Items for Systematic Reviews and Meta-Analyses) [29]. It is important to note that this systematic review did not require ethical approval, as it involved the synthesis and analysis of existing published data and did not involve direct interaction with human subjects or the collection of new primary data. See S1 Checklist for PRISMA guidelines.

### Eligibility criteria

Eligibility was based on compliance with the criteria described below. A summary of these criteria is provided in S1 Table.

### Participants

This review targeted studies including participants five to 24 years old living with a disability of any kind (developmental, learning, emotional, physical, intellectual or sensory). The American Psychological Association (APA) defines disability as: “a lasting physical or mental impairment that significantly interferes with an individual’s ability to function in one or more central life activities, such as self-care, ambulation, communication, social interaction, sexual expression, or employment”[30]. Examples include autism spectrum disorder, Down syndrome, cerebral palsy, spina bifida, visual impairment/blindness, hearing impairment and mobility limitations. These criteria were selected to ensure heterogeneity within the population and the number of possible outcomes resulting from the PA programs regarding different disabilities. The age range was selected to encompass three distinct period of life at which PA plays a crucial role in development: childhood, adolescence, and young adulthood, thereby providing a broader perspective on the effects of PA programs.

### Intervention

The studies in this review examine the effects of participation in group-based PA or sports programs for youths with disabilities. Programs are defined as structured and organized sessions supervised by a paid or volunteer coach, adult, or specialist in any setting (outside, indoor, park, pool) and adapted for youth with disabilities to offer them an opportunity to have fun and practice PA in a safe and secure environment for a certain amount of time (i.e., 2 weeks or more). Thus, competitive, school-based, or performance-oriented interventions (i.e., those designed to meet institutional standards, benchmarks, or performance goals—such as academic grading systems, elite sports rankings, or skill certification programs) and therapy-based interventions (intended to relieve or heal a disorder) were excluded. These interventions typically require structured, individualized, or highly specialized approaches, which do not align with our focus on community-based programs designed for broader accessibility and inclusivity. The exclusion criterion was based on the need to examine interventions that can be implemented in non-specialized, everyday community settings without requiring professional oversight or predefined performance metrics. As well, this review focuses on programs and interventions that can be carried out by PA specialists or educators within the community since they are more accessible and can benefit more than one participant at a time.

Additionally, studies were not accepted if the intervention was conducted on a one-to-one basis or if the participant was paired with an instructor, parent or specialist, as the aim was to examine the effects of participation in a group-based environment (i.e., with other participants to promote social interactions). Indeed, while adult support can facilitate PA sessions, it can also hinder opportunities for autonomy and friendships, particularly in the case of youths with disabilities [31,32].

For the purposes of this review, therefore, group-based activity required at least three individuals sharing similar characteristics (e.g., age), given that a group is usually defined as “two or more people who interact with and exert mutual influence on each other” [33]. Thus, this review focuses on groups of three or more individuals to avoid pair sports that limit interaction during play (e.g., tennis, badminton). Accordingly, all individually-performed interventions or programs (e.g., swimming, surfing, training exercises, fitness, treadmills, walking) were excluded unless performed in a group setting involving exchange, play, games or any kind of social contact including recurring communication with other participants. Home-based interventions (home exercise programs, online exercises, device-served interventions (e.g., Wii console) were also excluded. The only programs selected were those requiring the participants’ physical presence.

Mixed intervention sessions involving an individual PA (other than warm-ups and cool-downs) first and in a group setting later (not necessarily in this order) were likewise excluded. Other unrelated, multi-component interventions (e.g., diet plans, meetings) (e.g., health programs) were also excluded, as the primary focus was the benefits of PA programs themselves and not the related components. Finally, programs that involved pairing participants without disabilities together with those with disabilities were included, however only the effects towards participants with disabilities were examined.

### Outcomes

The outcomes of this systematic review offer information regarding the effects of adapted PA programs on youth with disabilities. More specifically, it focusses on identifying variables that reflect the effects of group-based adapted PA program across four key domains: cognitive (e.g., attention, memory), affective (e.g., self-esteem, emotional well-being), physical (e.g., strength, endurance), and social (e.g., social interaction, communication). As well, they provide insights on PA programs characteristics including length, frequency, intensity, PA, trainers, adaptation details, group settings, implementation, limitations and recommendations for program development.

### Study design

The current review included quasi-experimental design and randomized controlled trials. Non-empirical studies, theses, meta-analyses, grey literature and reviews of any type were excluded.

### Search strategy

Articles related to group-based PA programs for youths (5–24 years old) were collected from the following databases: APA PsycINFO (498), Medline (1396), SPORTDiscus (541), and Érudit (280). Each database was searched using the same keywords (translated into French for Érudit, as it is a French-language database), with the search strategy consistently applied across all platforms as we used subject headings (SU) to ensure comprehensive coverage, including MeSH, Thesaurus, and other controlled vocabulary terms specific to each database (see Table 1 and S1 List). All query terms were individually verified for compatibility, confirming that the search equation was uniformly effective for the following categories generated with the assistance of a specialized librarian: 1) Program, 2) Physical activity, 3) Disabilities, 4) Youth (5–24 years old). Finally, potentially eligible studies were also selected through a manual search of the studies’ reference lists targeting specific disability (See S2 List) and through forward citation tracking (via all previous databases, PubMed and Google Scholar) of the articles included in this systematic review. Searches were conducted from June 6, 2022 to January 15, 2025.

**Table 1:** Search strategy.

1. Program	TI (“Program*” OR “Intervention*”) OR SU (“Program*” OR “Intervention”) OR AB (“Program*” OR “Intervention”)
2. Physical activity	TI (“Physical activity” OR “Sport” OR “Exercise”) OR SU (“Physical activity” OR “Sport” OR “Exercise”) OR AB (“Physical activity” OR “Sport” OR “Exercise”)
3. Disabilities	TI (“Disab*” OR “Special need” OR “Sensory disability” OR “Physical disability” OR “Physical disorder” OR “Learning disability” OR “Communication disorder” OR “Language disorder” OR “Developmental disorder” OR “Intellectual disability” OR “Behavior disorder” OR “Emotional difficulty” OR “Social difficulty” OR “Cognitive difficulty”) OR SU (“Disab*” OR “Special need” OR “Sensory disability” OR “Physical disability” OR “Physical disorder” OR “Learning disability” OR “Communication disorder” OR “Language disorder” OR “Developmental disorder” OR “Intellectual disability” OR “Behavior disorder” OR “Emotional difficulty” OR “Social difficulty” OR “Cognitive difficulty”) OR AB (“Disab*” OR “Special need” OR “Sensory disability” OR “Physical disability” OR “Physical disorder” OR “Learning disability” OR “Communication disorder” OR “Language disorder” OR “Developmental disorder” OR “Intellectual disability” OR “Behavior disorder” OR “Emotional difficulty” OR “Social difficulty” OR “Cognitive difficulty”)
4. Population (Youth)	TI (“Child” OR “Teenager” OR “Adolescent” OR “Youth” OR “Young adult”) OR SU (“Child” OR “Teenager” OR “Adolescent” OR “Youth” OR “Young adult”) OR AB (“Child” OR “Teenager” OR “Adolescent” OR “Youth” OR “Young adult”)
5. 1 AND 2 AND 3 AND 4	

*Note.* TI = title; SU = subject terms; AB = abstract; Limiters are: Years of publication: 2013-2025; Age groups: preschool to young adulthood; Languages: English or French

### Study selection

**Fig 1:**
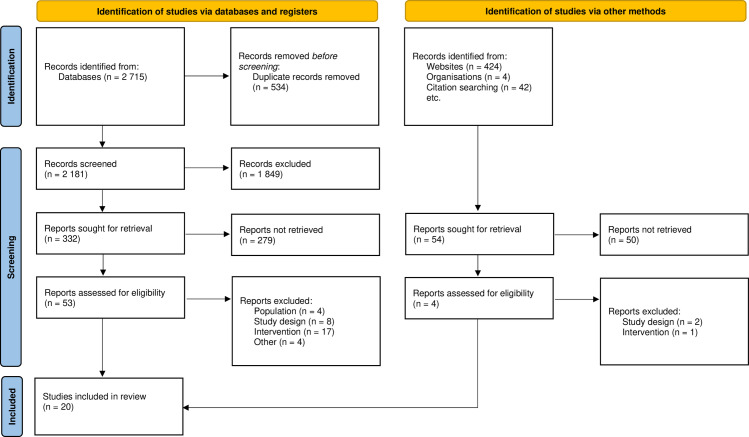
Prisma flow chart.

After the search of the different online databases was completed, two authors reviewed the titles and abstracts of 2,181 articles and independently selected those satisfying the eligibility criteria. After the selection was validated, 332 articles were retrieved. Both authors rescreened these articles, which led to 53 articles assessed for eligibility. The data of the 53 selected articles were extracted after a full reading of the text and the completion of an extraction grid. A total of 33 articles were excluded for different reasons related to eligibility criteria (see S1 Data). If doubts remained about certain articles, a third author was consulted to make the final decision. Overall, 19 articles were selected for this review through an automated database search, and an additional manual search found one study to be eligible. A total of 20 studies are included in the present review (see Fig 1).

### Risk of bias assessment

The quality of the studies was assessed using the RoB 2 tool (Risk of Bias 2) for randomly assigned interventions and the ROBINS-I tool (for assessing risk of bias in non-randomized studies of interventions) for non-randomly assigned designs, since this review used experimental and quasi-experimental designs. The RoB 2 tool is a revised Cochrane risk-of-bias tool specifically designed to evaluate the risk of bias in randomized trials. It covers domains including bias from the randomization process, deviations from intended interventions, missing outcome data, measurement of outcomes and selection of reported results [34]. The ROBINS-I tool, on the other hand, is designed to assess the risk of bias in non-randomized studies of interventions and includes domains such as confounding variables, selection of participants, classification of interventions, deviations from intended interventions, missing data, measurement of outcomes and selection of reported results [35].Two reviewers independently assessed the methodological quality of the studies included using the criteria for the study design. Disagreements over risk of bias assessments or the rationales behind these assessments were resolved through deliberation and dialogue. Next, an overall summary risk of bias (ROBINS-I: low, moderate, serious, critical; RoB 2: low, some concerns, high risk) was generated for each outcome, where the study’s overall bias level was determined based on the highest level of bias identified among the different domains.

### Data extraction and analysis

For all selected studies or articles, information was retrieved using a grid that included the following variables: country, participants, study design, programs’ components, study outcomes, study limitations and recommendations. All variables were analyzed in quantitative terms to obtain an overview of the studies’ characteristics. Next, the studies’ main themes were identified and compared in terms of the results of each study. The main themes, which were then grouped together, are discussed in the results section of this review.

## Results

A total of 20 studies met the inclusion criteria. The primary outcomes from the selected studies highlight mainly physical and social advantages. The list of the included studies is in S3 List.

### Study characteristics

Most of the studies were conducted in the United States (*n* = 6) and Serbia (*n* = 4). Additionally, a single study was conducted in each of the following countries: the United Kingdom, China, Canada, Greece, India, Italy, Spain, Taiwan and South Korea. Regarding sample size, samples ranged from six to 83 participants and included mainly boys (average ratio of 4:1; boys: girls). The age of participants varied from five to 24 years old, although the vast majority presented a mean age of approximately 10 years old. The disabilities most often identified in the studies were intellectual disability (ID; *n* = 11), autism spectrum disorder (ASD; *n *= 8) and Down syndrome (DS; *n *= 4). Other disabilities included cerebral palsy (CP; *n* = 1); unspecified physical disability; *n* = 1; visual impairment (VI; *n* = 1); fragile X (FXS; *n* = 1); fetal alcohol spectrum disorder (FAS; *n* = 1) and developmental delay (DD; *n* = 1). Of the 20 articles, two examined participants with combined disabilities (e.g., ASD and ID, often referred to as IDD) [36,37]. Two other researches included participants with different disabilities in their intervention group (e.g., ASD, ID, DS, CP) [38,39]. For further details, see S2 Table.

### Program description

Overall, the length of the programs varied from four to 24 weeks. Programs were held from one to five times per week and lasted 30–90 minutes. PA was presented in various forms. Programs or interventions were often divided into sequences: warm-ups (5–10 mins), practice and/or main activity (20–45 mins each) and a cool-down period (5–10 mins). In some cases, progressions between sessions were also used (e.g., gradually more practice time). For further details, notably regarding stations or the specific evolution of the intervention, see S3 Table. The most popular PAs were soccer (*n* = 4) and martial arts (*n* = 4; judo = 2), karate, mixed). Other PAs were basketball (*n* = 2), gymnastics, community running, floor hockey, group drumming, yoga, football and climbing (*n* = 1 for each of these physical activities). The remaining studies (*n *= 4) included various PAs offered as multiple stations allowing the participants to navigate their session [36–39,40].

### Trainers

Trainers involved in the interventions were highly diversified, and included instructors, professionals, teachers, physical education teachers, trainers, therapists, physiologists and coaches. All had the skills, experience, training, or certification required for leading the programs. Of the 20 studies, only three proposed additional instruction for trainers, which consisted of a course assisted by an expert [41], a previous involvement in a pilot study [36] and some mentoring with a coach [42]. Additionally, the numbers of trainers vary significantly between interventions, and information was sometimes missing regarding the ratio between trainers and participants. For further details, see S3 Table.

### Group settings

Group settings and composition were often described as subgroups of six to eight participants. Three studies include pairing with a neurotypical participant [43–45]. Unfortunately, eight studies fail to provide information about group settings; descriptions of the interventions, however, the information available in those studies indicates that the activities were performed with all the participants [37–40,42,46–50]. Additionally, the authors of these studies (*n* = 8) offer further details including the involvement of games, sports or group activities. As well, all participants performed the activity in small groups similarly to the rest of the studies (*n* = 12) selected for this review.

### Adaptation details

Multiple adaptations are described in the 20 studies selected. However, it should be pointed out that six of the 20 studies do not propose any adaptations through their program setting. For all details regarding program adaptations, see S4 Table. Among these adaptations, two studies [38,51] stress the importance of initiating the program by familiarizing participants with routines and equipment. Similarly, Angeli et al. [51] and Hsu et al. [42] highlight participants’ autonomy by selecting specific training adaptable to individual needs, thus depicting a participant-centered approach. Stojanović et al. and Ekins et al. [40,46], for their part, focus on participants’ emotional states. Consistent with this emotional aspect, Phung et al. [52] propose strategies for emotional regulation with reminders to use breathing techniques. Alternatively, Angeli et al. [51] and Hsu et al. [42] use additional practice activities to increase skills via different trainers, echoing the approach of a 1:3 coach-to-child ratio proposed by Collins et al. [39]. In contrast, Mohanty et al. [53] provide detailed instructions through audio cassettes. Ryuh et al. [45] argue for disability awareness education through neurotypical pairing for cooperative environments and prejudice reduction. In terms of environmental considerations, Morales et al. [47] and Pierantozzi et al. [49] call for large, well-ventilated spaces for participant safety, while Chen et al. [44] focus on weekly indoor training. Finally, methodological rigour was strengthened through previous pilot studies, which was the case for the pre-experiment validations of Choi et al. [36] and Xu et al.’s [37] as regards their chosen interventions.

### Outcomes

The programs’ outcomes show that they positively influence multiple aspects of the well-being of youths with disabilities, most especially, physical and motor skills, social and communication skills, affective and behavioral skills, and cognitive skills. See S5 Table for more details.

### Physical and motor skills

Eleven studies demonstrate that PA interventions can improve motor skills and physical fitness in individuals with disabilities. First, soccer and basketball training for ID and DS, and climbing for ASD, reveal a common trend of enhancing motor skills, while floor hockey and judo interventions also contribute to motor proficiency and balance improvement [38,40,42,43,48,50,54,55]. Second, gymnastic, drum training, and yoga interventions collectively showed improvements in muscle strength and overall fitness across diverse disabilities (ASD, ID, VI, DS) [37, 46,53]. Finally, PA, including sports and judo, leads to improved cardio-metabolic health and cardiorespiratory fitness for young individuals with ASD [39,49].

### Social and communication skills

Six studies examine the social effects of group-based PA programs. Two of these reveal that a judo and karate group-based program positively affected social interactions, social communication and social skills for young people with ASD [41,47]. Another program mixing ASD and neurotypical participants through inclusive soccer found that inclusive settings enhance positive contacts and experiences [45]. Hsu et al. [42], furthermore, found that a floor hockey program benefited adaptive development and that participation in a multimodal PA program generated psychosocial development benefits for youths with ID. Finally, Periç et al. [54] highlight how soccer training can enhance social behaviour among young individuals with DS [36].

### Affective and behavioural skills

Six studies examine the affective and behavioural impact of group-based PA programs. Indeed, one community-run program for youth with physical disabilities led to improvements in self-concept in terms of scholastic competence, athletic competence and physical appearance [51]. A judo program also improved the emotional responses of ASD participants in a social context [47]. Similarly, a program implementing PA stations observed improvement in emotional self-control following PA in both training and classroom contexts [36]. The mixed martial arts intervention described in the study by Phung et al. [52] strengthened behavioural and emotional regulation for youths with ASD. Finally, Periç et al. [54] discovered that their adapted soccer training can reduce aggression, anxiety and depression levels in young DS individuals.

### Cognitive skills

Three articles explore the effect of group-based PA programs on cognitive function. A study by Chen et al. [44] found that a soccer program could improve reaction time and executive function among young ID adults. Another by Phung et al. [52] suggests that a mixed martial arts program appears to promote the use of executive functions such as behavioural inhibition and working memory. Finally, Periç et al. [54] demonstrate that their soccer program improved the attention of DS participants.

### Combined skills

Six studies survey the effect of group-based PA programs on combined outcomes [36,38,42,47,52,54]. The benefits of their interventions or programs had repercussions in more than one of the areas mentioned above. Indeed, two programs impacted participants’ overall quality of life (functioning, physical health, emotional well-being) [38,47]. One program, moreover, affected all areas and demonstrated the effectiveness of adapted soccer training in reducing aggression, anxiety, and depression levels, while enhancing attention, social behaviour, and fundamental motor skills [54].

### Risk of bias assessment

Most of the articles included in this review either fail to indicate limitations or mention similar ones: small simple size, inequality of gender distribution, no replicable measurement or task, and lack of information regarding long-term effects. Only two studies examine the long-term effects of their interventions [41,47]. Thus, it was not possible to generalize results or replicate all programs. In addition, the presence of confounding variables (e.g., previous experience in PA, initial skills levels, severity of disabilities, additional treatments outside the intervention, motivation, and mood change) can influence the quality of the study and the validity of results.

This being said, almost all studies were considered to have some concerns or a serious risk of bias following use of the risk of bias tools (RoB 2 and ROBINS-I; see S6 Table). Additionally, certain variables were not assessed or could have been assessed with better tools as reported in multiple studies. The authors of only one study [52], in fact, discuss their program’s fidelity of implementation, which ranged from 73 to 100%. Another issue is that parents were often asked to complete questionnaires because the child could not complete the surveys. Thus, the authors were unsure as to whether the parents’ perceptions were the same as those of their child regarding the latter’s progress while participating in the programs and hence questioned the value of the results. Finally, while most of the studies employ a quasi-experimental design (*n* = 16), only 11 include control or comparison groups.

## Discussion

This systematic review aimed to identify the characteristics and outcomes of group-based PA programs for young people (5–24 years old) with disabilities. A total of 20 studies proposed programs with group-based physical activities corresponding to the selection criteria. The results from the studies selected indicate that young people with disabilities could see improvements in various aspects such as physical, social, affective, and cognitive ones. Moreover, this review clarified the characteristics of group-based PA programs by identifying strategies for effectiveness, which represents an improvement over previous researches (see S7 Table for a summary of all extracted data). Still, some methodological issues remain and recommendations regarding these challenges will be offered to guide future research.

### Program characteristics

The components and characteristics of the programs vary significantly, but all propose interesting ways to better understand the strengths and limitations of group-based PA programs.

### Program structure

Most of the time, the frequency, intensity, and length of the programs were, respectively, twice a week, 30–60 minutes, and for a 12-week period. As a previous study [56] shows, children with certain disabilities can participate in group-based programs that include weekly sessions of 30–60 minutes. Accordingly, it may be advisable to respect these parameters, as anything more demanding may be highly energy consuming and require significant resources and adaptation time. The current review, moreover, reveals that at least 30 minutes of PA per week in a PA program can yield benefits for youths with disabilities. According to the literature [57], people living with disabilities can derive health benefits from an average of 20 minutes per day of PA. These findings highlight the importance of encouraging consistent participation in PA, even in smaller amounts, as it can contribute significantly to the overall health of youths with disabilities. Otherwise, the intensity, frequency, and duration of activities in the selected studies did not necessarily align with the significance of the outcomes. This shows there is no clear evidence that greater intensity, greater frequency, or longer practice sessions result in more meaningful outcomes.

These findings are in line with those of a previous systematic review examining the effectiveness of group-based organized participation in PA for children with ASD [27]. The Howells review suggests that determining the optimal dosage for such programs require more attention as even shorter programs had shown benefits. Otherwise, the findings of the research conducted by Haegele et al. [58] propose that the demeanor of the trainer and PA environment, including peers and settings, exert significant influence as facilitators for PA involvement. These later results suggest that the surrounding environment and individuals involved in PA are more significant than the specific program parameters in achieving favorable outcomes for youth with disabilities.

### Content and features of physical activity

The most popular programs proposed were soccer and martial arts. Indeed, these two PAs require little equipment and can be practiced conveniently in multiple settings, which makes them easier to implement. Nevertheless, they call for some form of training or at least PA-related experience. As observed in the studies examined here, all trainers in the programs were qualified in their domain-specific PA. Qualification appears to be an important factor for leading a program of this kind, which may explain why very few interventions in this review require additional training for trainers. One aspect the selected studies fail to mention is the significance of the trainers’ connection to the participants and how this impacts the program’s effectiveness. The literature on this subject suggests that diversified trainers and the presence of specific characteristics (e.g., a positive attitude) may enhance the PA experience [59].

As regards group settings, pairing neurotypical participants with neurodivergent participants seems to be a good alternative for facilitating progress and evolution through PA programs, and other studies demonstrate the effectiveness of this strategy [4,60,61]. Participants are often divided into subgroups of six to eight, however little information is given as to why and how these subgroups are created. Smaller groups are no doubt easier to manage, but further evidence is needed regarding use of this procedure with youths with disabilities in group-based PA programs. Additionally, other reviews report that there is no information on group composition and the reasons programs are implemented [14]. Another important point is that very few studies discuss youths with combined disabilities or with different disabilities in the same groups. As reported in other studies, it may be more difficult to incorporate heterogeneous groups as this involves more staff, more needs to fill and generally more preparation [14,15]. However, implementing such groups could promote inclusivity, be less experiment-driven and propose new avenues for PA programs in community settings.

### Strategies for effectiveness

Although previous researches highlight the lack of information on adaptation details or program process [14], multiple specifications have emerged in recent years regarding programs’ strategies and adaptations. Indeed, several programs include adaptation time (gradual contact with environment and intervention), which can help participants to become familiar with settings and equipment [38,51]. Trainers can therefore react, rearrange and adapt the environment based on participants’ special needs. By identifying individual needs, the program manager can consider the emotional state, current mood and interests of the participants [40,42,46,51,53], so that the proposed intervention better matches the participants’ abilities. Moreover, the support of trainers and the implementation of a ratio per participant are used to prevent complications [39].

The results of this review also highlight a lack of information for addressing crisis situations. Indeed, only one study addressed solutions to frustration or overwhelming crisis other than parents’ involvement [52]. Thus, a specific protocol should be developed on how to handle participants’ frustration behaviours or overwhelming emotions during sessions. For example, because individuals with ASD are very sensitive to their surroundings [62], the ability to properly address, react and respond to this sensitivity limits the potential harm to both the participants and their peers. An alternative is to adapt the environment to youths with disabilities to make it safe, adequate and well spaced [47,49]. One article describes a pilot study conducted to validate their intervention [36]. Others used a progression sequence as the session advanced [37,44]. For example, time was added throughout the sessions as the participants became accustomed to the intervention.

### Methodological concerns

An important limitation of group-based activity programs is confounding variables. Indeed, when studying youths with disabilities, several variables liable to impact the validity of the results must be controlled. The main confounding variables identified in this review include previous PA experience, initial skills level, severity of disabilities, additional treatment outside the intervention, motivation, and mood change. The risk of bias within all studies is therefore relatively high. The reason may be that in most cases, the assessor or participants were aware of the conditions of the experiment. Also, the fact that parents are frequently asked to complete questionnaires or take part in data collection often reduces the validity of the results. Finally, there is a need for programs to conduct fidelity of implementation measures before evaluating their outcomes. As reported in this review, only one study discussed fidelity of implementation [52]. Hence, empirical value should be promoted through the assessment of fidelity as it could subsequently facilitate measurements and data collection. As well, the assessment of fidelity for group-based PA programs would provide a main framework that could benefit other organizations and be employed in multiple communities.

### Outcomes

This section discusses the implications of the outcomes retrieved from the programs, highlighting their significance and potential impact on future developments.

### Physical outcomes

Over half the studies in this review examine physical outcomes (*n* = 11). Results indicate that group-based PA programs for youths five to 24 years old can improve general physical fitness, specific motor skills, aerobics, strength, coordination, balance and endurance. Indeed, according to a review of reviews [14], most other studies on PA programs report physical outcomes as the main results. Similarly, results from the current review indicate that PA programs benefit the physical development of youths with diverse disabilities. While this review does not cover all disabilities, the results show multiple benefits for some specific types including a few, like Fragile X syndrome, which are less common.

### Social and affective outcomes

The studies in this review reveal the positive effects of group-based PA programs on young people’s social and affective development. The main social outcome for youths with disabilities is improved emotional responses in areas such as self-control and emotional regulation. Additionally, results suggest that group-based PA programs help reduce anxiety and symptoms of depression [54], which is consistent with findings elsewhere in the literature [63,64]. Nonetheless, the mechanisms behind these social and affective improvements, however, are not fully described. Most of the studies reviewed lack detailed information on the underlying processes of social skills development, making it difficult to discern specific meanings within a given context due to numerous confounding variables. Still, the studies included in this review show that being part of a community or a group facilitates communication opportunities and offers youths with disabilities a plausible option for developing social skills [36,41,42,45,47,54].

These findings are in line with previous studies that promote the importance of organized, group-based PA participation for the social development of young people with disabilities [17,26,27,65]. However, it should be noted that most studies on social outcomes in this review discuss youths with ASD and ID. Nevertheless, the results are highly promising, given that multiple studies of youths with different disabilities (e.g., DS) show that social capabilities are enhanced through PA programs.

### Cognitive outcomes

Regarding cognitive outcomes, this review’s main finding is that group-based PA programs can improve reaction time, inhibition and attention [44,52,54], indicating that PA can be beneficial in other settings such as schools. Interestingly, another review suggests similar results [66]. Yet, cognitive outcomes remain the less studied domain for group-based PA programs among youth with disabilities as it is often the case in PA research [14]. Assessing cognitive achievements regarding young individuals with disabilities presents a considerable problem for researchers for two reasons. First, the broad range of disabilities includes diverse cognitive capacities and constraints, making the use of standardized evaluation instruments challenging [67,68]. Next, the intervention aside, cognitive development is influenced by a complex interaction of genetic, environmental and individual factors. In short, the variety of disabilities together with the limitations of conventional assessment tools add to the difficulty of measuring cognitive outcomes for youths with disabilities in research studies [68,69].

### Recommendations for future studies

Future studies should focus on understanding the possible differences between high-incidence disabilities (e.g., ASD and ID) and low-incidence disabilities (e.g., Fragile X syndrome). Additionally, samples should include more female participants. Because cognitive outcomes are least discussed in terms of interventions, further research is needed in this area, given that cognitive skills can often be transferred to other areas of life. Moreover, further investigations are necessary to identify the specific mechanisms behind outcomes (e.g., key moments, specific cerebral zone activity, specific behaviour patterns, etc.). Other recommendations include employing additional adapted measures, considering past sport experiences, increasing sample size, and ensuring that programs are feasible and realistic (e.g., costs are kept low). It was also suggested to consider participants’ interests and ensure a safe, suitably equipped environment that promotes a balance between fun and development [42,47,49,51]. More programs need to identify clear protocol and crisis management strategies for youths with disabilities, and the number of confounding variables should be addressed to limit bias. Similarly, the impacts of trainers should be considered while measuring fidelity of implementation and desired outcomes. The long-term effects of these programs thus require further study, as evidence of sustainability is missing for most interventions.

More information is also required to better understand group dynamics, including composition. As well, further studies need to examine the possibilities for group-based practice to include heterogeneous participants, which could promote inclusivity and facilitate the implementation of community programs. More action is also necessary to ensure greater accessibility to PA programs for youths with disabilities. Overall, the studies included in this review suggest that group-based PA programs have significant potential when it comes to these individuals and should be implemented in parallel with special schools or treatment. The recommendations derived from these selected studies can therefore guide future research and program development.

### Limitations

This review adhered to several criteria and used a large initial sample size. A major effort was made to document the procedures employed (PROSPERO; CRD42023392682) clearly and comprehensively. However, the eligibility criteria (e.g., exclusion of therapy-based, school-based, home-based and group-based programs) were very specific, and at times the lack of details in certain studies made classification difficult. A result is that some interesting studies may have been left out. However, in cases of uncertainty, the studies were carefully examined and analyzed before being excluded, and reasons for the decision were provided. One limitation of this review is the exclusion of qualitative studies. The reason, however, is that our primary focus was on experimental and quasi-experimental designs presenting quantitative data offering crucial insights into the studies’ empirical validity. A second limitation is that some disabilities were not represented in the 20 selected articles.

## Conclusions

In summary, this systematic review aimed to clarify the characteristics and outcomes of group-based PA programs for individuals with disabilities aged five to 24 years. The review not only shed light on program characteristics but also identified strategies for program effectiveness, marking a significant step forward from previous researches. Methodological challenges persist nevertheless, and recommendations are made to address these problems in future research. Despite this review’s limitations, its findings endorse the implementation of group-based PA programs to promote holistic development and well-being among youths with disabilities. The insights gathered in this review offer valuable guidance for researchers, program creators and practitioners seeking to develop an effective group-based PA program for these individuals.

## Supporting information

S1 List Search strategy details. (DOCX)

S2 List Handsearch details. (DOCX)

S3 List Included studies. (DOCX)

S1 Table Inclusion and exclusion criteria. (DOCX)

S2 Table General description of the programs. (DOCX)

S3 Table Programs settings. (DOCX)

S4 Table Adaptation details. (DOCX)

S5 Table Programs outcomes. (DOCX)

S6 Table Risk of bias. (DOCX)

S7 Table Summary of extracted data. (DOCX)

S1 Checklist Prisma checklist. (DOCX)

S1 Data Selection process. (XLSX)
