# Supplementary material for: Effects of group-based physical activity programs on children, adolescents, and young adults with disabilities: A systematic review
Source: PLoS One. 2025 May 23;20(5):e0323707. doi: 10.1371/journal.pone.0323707 (PMC12101651; doi:10.1371/journal.pone.0323707)
Supplement: S1 List — (DOCX) [file pone.0323707.s001.docx]

**S1 List. Search strategy details.**

The following search strategies were developed with the support of a professional librarian and applied consistently across databases: APA PsycINFO, SPORTDiscus, Medline and Érudit. Boolean operators, database-specific subject headings (e.g., MeSH terms in Medline), and keyword combinations were applied to title (TI), subject (SU), and abstract (AB) fields to ensure comprehensive and replicable results across all databases. The inclusion of the subject (SU) field allowed us to capture controlled vocabulary and indexing terms that may not have been explicitly stated in the title or abstract but were relevant to the topic—particularly for identifying a broader range of disabilities. The abstract (AB) and title (TI) fields were prioritized to focus the search on studies whose core content aligned with the review objectives, while still ensuring a sufficient breadth of coverage given the high volume of literature regarding the term (disabilities).

The Boolean equation was **intentionally designed to be flexible and database-compatible** by relying primarily on **free-text terms** that also correspond to terms found in the controlled vocabularies of each database:

- In **MEDLINE**, many of the terms matched **MeSH** headings (e.g., *Exercise*, *Disabled Persons*, *Adolescent*).
- In **APA PsycINFO**, terms aligned with the **APA Thesaurus** (e.g., *Physical Activity*, *Disabilities*, *Youth*).
- In **SPORTDiscus**, the same terms were found as **Subject Terms**.
- In **Érudit**, which lacks a standardized controlled vocabulary, the same terms were translated into French and used as free-text keywords.

Before finalizing the equation, each term was **individually tested** in its respective database to confirm its compatibility with that database’s indexing system and its return of relevant and sufficient results

This process allowed us to use **one single, replicable equation** across all platforms, minimizing bias and maximizing reproducibility while respecting the structure of each database.

***MAIN EQUATION***

**TI ("Program*" OR "Intervention*") OR SU ("Program*" OR "Intervention") OR AB ("Program*" OR "Intervention")**

**TI ("Physical activity" OR "Sport" OR "Exercise") OR SU ("Physical activity" OR "Sport" OR "Exercise") OR AB ("Physical activity" OR "Sport" OR "Exercise")**

**TI ("Disab*" OR "Special need" OR "Sensory disability" OR "Physical disability" OR "Physical disorder" OR "Learning disability" OR "Communication disorder" OR "Language disorder" OR "Developmental disorder" OR "Intellectual disability" OR "Behavior disorder" OR "Emotional difficulty" OR "Social difficulty" OR "Cognitive difficulty") OR SU ("Disab*" OR "Special need" OR "Sensory disability" OR "Physical disability" OR "Physical disorder" OR "Learning disability" OR "Communication disorder" OR "Language disorder" OR "Developmental disorder" OR "Intellectual disability" OR "Behavior disorder" OR "Emotional difficulty" OR "Social difficulty" OR "Cognitive difficulty") OR AB ("Disab*" OR "Special need" OR "Sensory disability" OR "Physical disability" OR "Physical disorder" OR "Learning disability" OR "Communication disorder" OR "Language disorder" OR "Developmental disorder" OR "Intellectual disability" OR "Behavior disorder" OR "Emotional difficulty" OR "Social difficulty" OR "Cognitive difficulty")**

**TI ("Child" OR "Teenager" OR "Adolescent" OR "Youth" OR "Young adult") OR SU ("Child" OR "Teenager" OR "Adolescent" OR "Youth" OR "Young adult") OR AB ("Child" OR "Teenager" OR "Adolescent" OR "Youth" OR "Young adult")**

***APA PsycINFO***

| **Term Used** | **Controlled Vocabulary Match** | **Respective Equivalent or Related Term(s) of interest from the database** | **Concept** |
| --- | --- | --- | --- |
| Program* | APA Thesaurus | “Program Development or Program evaluation or Services or Interventions” | Program / Intervention |
| Intervention* | APA Thesaurus | “Early Intervention or Family Intervention or Group Intervention or Prevention or Crisis Intervention Services or At Risk Populations or Program or Services or Best Strategies” | Program / Intervention |
| Physical activity | APA Thesaurus | “Motor Processes or Exercise or Active Living or Activity Level or Health Behavior or Kinesiology or Locomotion or Motor Control or Physical Fitness or Sport and Exercise Measures or Wearable Devices” | Physical Activity |
| Sport | APA Thesaurus | “Recreation or Adaptive Sports or Athletes or Athletic Participation or Athletic Performance or Baseball or Basketball or College Sports or Cycling or Extreme Sports or Football or High School Sports or Judo or Martial Arts or Professional Sports or Soccer or Sports Coaching or Swimming or Tennis or Weightlifting or Athletic and Sports Personnel or Athletic Training or Coaches or College Athletes or Professional Athletes or Sport and Exercise Measures or Sport or Teams or Wilderness Experience…” | Physical Activity |
| Exercise | APA Thesaurus | “Physical Activity or Physical Exercise or Aerobic Exercise or Weightlifting or Yoga or Body Weight Cycling or Health Behavior or Kinesiology or Physical Fitness or Sport and Exercise Measures or Sport and Exercise Psychologists or Sport Psychology or Wearable Devices or Weight Control” | Physical Activity |
| Disab* | APA Thesaurus | “Disorders or Developmental Disabilities or Learning Disabilities or Multiple Disabilities or Reading Disabilities or Accessibility (Disabilities) or Accommodation (Disabilities) or Adaptive Sports or Disability Discrimination or Disability Evaluation or Disability Inclusion or Disability Management or Disability Services or Mental Disorders or Physical Disorders or Handicaps or Physical Disabilities or Exceptional Children (Handicapped)…” | Disability / Special Needs |
| Special need | APA Thesaurus | “Needs or Disorders or Early Intervention or Inclusive Education or Individual Education Programs or Mental Disorders or Needs Assessment or Physical Disorders or Response to Intervention or Special Education or Special Education Students or Transition Planning” | Disability / Special Needs |
| Sensory disability | Not controlled, free text only | - | Disability / Special Needs |
| Physical disability | Not controlled, free text only | “Disabled or Mobility impairment or Handicap” | Disability / Special Needs |
| Physical disorder | APA Thesaurus | “Disorders or Cardiovascular Disorders or Congenital Disorders or Digestive System Disorders or Endocrine Disorders or Genetic Disorders or Health Impairments or Injuries or Lesions or Metabolism Disorders or Musculoskeletal Disorders or or Nervous System Disorders or Nutritional Deficiencies or Physical Disfigurement or Respiratory Tract Disorders or Sensory System Disorders or Skin Disorders or Vision Disorders or Back Pain or Chronic Illness or Chronicity (Disorders) or Communication Disorders or Comorbidity or Diagnosis or Disabilities or Eating Disorders or Illness Behavior or Memory Disorders or Mental Disorders or Pain or Special Needs or Symptoms or Syndromes…” | Disability / Special Needs |
| Learning disability | Not controlled, free text only | “Learning difficulties or Learning disorders” | Disability / Special Needs |
| Communication disorder | APA Thesaurus | “Nervous System Disorders or Hearing Disorders or Language Disorders or Social Communication Disorder or Augmentative Communication or Communication Skills or Developmental Disabilities or Language Therapy or Mental Disorders or Physical Disorders or Speech Anxiety or Speech Language Pathology or Speech Therapy” | Disability / Special Needs |
| Language disorder | APA Thesaurus | “Communication Disorders or Agnosia or Agraphia or Dyscalculia or Dyslexia or Echolalia or Mutism or Specific Language Impairment or Speech Disorders or Language Delay or Language Development or Neurolinguistics or Speech Language Pathology” | Disability / Special Needs |
| Developmental disorder | Not controlled, free text only | “Developmental disabilities” | Disability / Special Needs |
| Intellectual disability | APA Thesaurus | “Intellectual Development Disorder or Neurodevelopmental Disorders or Anencephaly or Crying Cat Syndrome or Down's Syndrome or Tay Sachs Disease or Adaptive Behavior or Adaptive Behavior Measures or Brain Damage or Cognitive Impairment or Developmental Disabilities or Fetal Alcohol Syndrome or Fragile X Syndrome or Hydrocephalus or Klinefelters Syndrome or Mental Disorders or Microcephaly or Phenylketonuria or Prader Willi Syndrome or Rett Syndrome or Savants or Williams Syndrome or Borderline Mental Retardation or Educable Mentally Retarded or Mild Mental Retardation or Moderate Mental Retardation or Oligophrenia or Profound Mental Retardation or Psychosocial Mental Retardation or Severe Mental Retardation or Slow Learners or Trainable Mentally Retarded” | Disability / Special Needs |
| Behavior disorder | APA Thesaurus | “Mental Disorders or Conduct Disorder or Disruptive Behavior Disorders or Emotional and Behavioral Disorders or Impulse Control Disorders or Oppositional Defiant Disorder or Pyromania or Self-Destructive Behavior or Acting Out or Aggressive Behavior or Antisocial Behavior or Behavior or Behavior Problems or Body Rocking or Crime or Criminal Behavior or Externalizing Symptoms or Faking or Fecal Incontinence or Kluver Bucy Syndrome or Symptoms or Thumbsucking or Trichotillomania” | Disability / Special Needs |
| Emotional difficulty | Not controlled, free text only | “Emotional Disturbances or Emotional Eating  or Emotional Exhaustion” | Disability / Special Needs |
| Social difficulty | Not controlled, free text only | - | Disability / Special Needs |
| Cognitive difficulty | Not controlled, free text only | - | Disability / Special Needs |
| Child | Not controlled, free text only | “Children or Adolescents or Youth or Teenager” | Age Group |
| Teenager | Not controlled, free text only | “Adolescents or Young adults” | Age Group |
| Adolescent | Not controlled, free text only | “Teenagers or Young adults” | Age Group |
| Youth | Not controlled, free text only | “Adolescents or Young people or Teen or Young adults” | Age Group |
| Young adult | Not controlled, free text only | “Adolescents or Teenagers or College students” | Age Group |

**Specific Limiters: Age groups: School Age (6-12) – Adolescence (13-17) – Young adulthood (18-29); Exclude Dissertations; Language: English – French**

***Medline***

| **Term Used** | **Controlled Vocabulary Match** | **Respective Equivalent or Related Term(s) of interest from the database** | **Concept** |
| --- | --- | --- | --- |
| Program* | MeSH | “Program Evaluation or Scope or Program Development or Services or Interventions” | Program / Intervention |
| Intervention* | Not controlled, free text only | “Program Evaluation or Scope or Program Development or Services or Interventions” | Program / Intervention |
| Physical activity | Not controlled, free text only | “Exercise or Fitness or Physical exercise Sport” | Physical Activity |
| Sport | MeSH | “Athletic Performance or Baseball or Basketball or Bicycling or Boxing or Camping or Cricket Sport or Dancing or Football or Gaelic Football or Golf or Gymnastics or Hobbies or Hockey or Leisure Activities or Martial Arts or Mountaineering or Play or Racquet Sports or Recreation or Rugby or Running or Skating or Snow Sports or Soccer or Sports or Sports for Persons with Disabilities or Team Sports or Track and Field or Volleyball or Walking or Water Sports or Weight Lifting or Wrestling or Youth Sports…” | Physical Activity |
| Exercise | MeSH | “Activities of Daily Living or Gymnastics or Human Activities or Movement or Muscle Stretching Exercises or Physical Conditioning or Post-Exercise Recovery or Running or Swimming or Walking or Warm-Up Exercise” | Physical Activity |
| Disab* | Not controlled, free text only | “Disability or Disabled or Impairment or Impaired or Special needs or Children with Disabilities or Developmental Disabilities or Developmental Disability or Disability Evaluation or Disability Studies or Intellectual Disability or Learning Disabilities or Persons with Disabilities or Persons with Hearing Disabilities or Persons with Intellectual Disabilities or Persons with Visual Disabilities or Professional Impairment” | Disability / Special Needs |
| Special need | Not controlled, free text only | “Disabilities or Intellectual disabilities or Developmental” | Disability / Special Needs |
| Sensory disability | Not controlled, free text only | “Children with Disabilities or Developmental Disabilities or Epilepsy or Hereditary Sensory and Autonomic Neuropathies or Intellectual Disability or Learning Disabilities or Persons with Disabilities or Persons with Intellectual Disabilities” | Disability / Special Needs |
| Physical disability | Not controlled, free text only | “Disabled or Mobility impairment or Handicap” | Disability / Special Needs |
| Physical disorder | Not controlled, free text only | - | Disability / Special Needs |
| Learning disability | MeSH | “Anxiety or Attention Deficit and Disruptive Behavior Disorders or Child Behavior Disorders or Child Development Disorders, Pervasive or Childhood-Onset Fluency Disorder or Communication Disorders or Developmental Disabilities or Intellectual Disability or Language Disorders or Learning Disabilities or Mental Disorders or Motor Skills Disorders or Mutism or Nervous System Diseases or Neurobehavioral Manifestations or Neurodevelopmental Disorders or Neurologic Manifestations or Pathological Conditions, Signs and Symptoms or Reactive Attachment Disorder or Social Communication Disorder or Specific Language Disorder or Specific Learning Disorder or Speech Sound Disorder or Stereotypic Movement Disorder or Tic Disorders…” | Disability / Special Needs |
| Communication disorder | MeSH | “Learning Disabilities or Language Disorders” | Disability / Special Needs |
| Language disorder | Not controlled, free text only | “Language impairment or Specific language disorder” | Disability / Special Needs |
| Developmental disorder | Not controlled, free text only | “Articulation Disorders or Communication Disorders or Developmental Disabilities or Dyslexia or Language Development Disorders or Learning Disabilities or Motor Skills Disorders or Psychomotor Disorders” | Disability / Special Needs |
| Intellectual disability | MeSH | “Mental retardation or Learning disability or Developmental disability or Learning disabilities or Cri-du-Chat Syndrome or De Lange Syndrome or Down Syndrome or Intellectual Disability or Prader-Willi Syndrome or Rubinstein-Taybi Syndrome or Trisomy 13 Syndrome or WAGR Syndrome or Williams Syndrome or X-Linked Intellectual Disability…” | Disability / Special Needs |
| Behavior disorder | MeSH | “Adverse Childhood Experiences or Bullying or Crime or Dangerous Behavior or Emotional Abuse or General Adaptation Syndrome or Homicide or Abuses or Illegitimacy or Incarceration or Incest or Incivility or Juvenile Delinquency or Runaway Behavior or Self-Neglect or Sex Work or Social Behavior Disorders or Social Problems or Social Sciences or Social Segregation or Sociology or Suicide or Underage Drinking or Violence…” | Disability / Special Needs |
| Emotional difficulty | Not controlled, free text only | “Emotional Abuse or Emotional Adjustment or Emotional Eating or Emotional Exhaustion or Emotional Regulation or Emotions or Expressed Emotion” | Disability / Special Needs |
| Social difficulty | Not controlled, free text only | - | Disability / Special Needs |
| Cognitive difficulty | Not controlled, free text only | “Cognitive dysfunction or Cognitive flexibility or Cognition disorder or Social cognition” | Disability / Special Needs |
| Child | MeSH | “Birth Cohort or Child, Preschool or Kids or Youth” | Age Group |
| Teenager | Not controlled, free text only | “Adolescents or Young adults” | Age Group |
| Adolescent | MeSH | “Teenagers or Young adults” | Age Group |
| Youth | Not controlled, free text only | “Young people or Teen or Young adults” | Age Group |
| Young adult | MeSH | “Middle aged or College students or Emerging adults” | Age Group |

**Specific Limiters: Age related: Child (6-12), Adolescent (13-18), Young adult (19-24); Language: English – French**

***SPORTDiscus***

| **Term Used** | **Controlled Vocabulary Match** | **Respective Equivalent or Related Term(s) of interest from the database** | **Concept** |
| --- | --- | --- | --- |
| Program* | Not controlled, free text only | “Services or Interventions” | Program / Intervention |
| Intervention* | Not controlled, free text only | “Strategies or Best practices” | Program / Intervention |
| Physical activity | Thesaurus | “Health or Exercise or Health Behavior or Physical Fitness or Physically Active People or Activity Level or Physical Activity Level or Exercise or Fitness or Physical exercise” | Physical Activity |
| Sport | Thesaurus | “AERONAUTICAL sports or AMATEUR sports or AQUATIC sports or ATHLETES or ATHLETIC clubs or ATHLETICS or BALL games or BALLISTICS in sports or BASEBALL or COLLEGE sports or COMBAT sports or COMMUNICATION in sports or CONTACT sports or CROSS-training (Sports) or DISC golf or DISCRIMINATION in sports or DOG sports or ENDURANCE sports or EXERCISE or EXTREME sports or Games or GYMNASTICS or HOCKEY or MOTORSPORTS or OLYMPIC Games or OUTDOOR life or PARKOUR or PHYSICAL education or PHYSICAL fitness or PHYSICS in sports or RACKET games or RECREATIONAL sports or ROLLER or SKATEBOARDING or SOFTBALL or SPORT for all or SPORTS for people with disabilities or SPORTS for youth or SPORTS psychology or SPORTS safety or SPORTS tournaments or SPORTSMANSHIP or STEREOTYPES in sports or TEAM sports or TEAMWORK (Sports) or VIOLENCE in sports or WINTER sports or WOMEN'S sports…” | Physical Activity |
| Exercise | Thesaurus | “PHYSICAL activity or PHYSICAL education or PHYSICAL exercise or PHYSICAL fitness or PHYSICAL training & conditioning or SPORTS or WARM-up or WARMUP or WORKING out or WORKOUTS (Exercise)” | Physical Activity |
| Disab* | Thesaurus | “COMMUNICATIVE disorders or DEVELOPMENTAL disabilities or DISABILITY or DISABLEMENT or DISABLING conditions or HANDICAP or HANDICAPS or HEARING disorders or IMPAIRMENT or MENTAL illness or MILD disabilities or MOVEMENT disorders or MULTIPLE disabilities or PEOPLE with disabilities or PERCEPTUAL disorders or PHYSICAL disabilities or PHYSICAL disability or PHYSICAL handicaps or PROSTHETICS or SEVERE disabilities or SEVERITY (of Disability) or VISION disorders” | Disability / Special Needs |
| Special need | Not controlled, free text only | “Special needs children or Children with disabilities” | Disability / Special Needs |
| Sensory disability | Not controlled, free text only | - | Disability / Special Needs |
| Physical disability | Not controlled, free text only | “Disabilities or Disabled or Mobility impairment or Handicap” | Disability / Special Needs |
| Physical disorder | Not controlled, free text only | - | Disability / Special Needs |
| Learning disability | Not controlled, free text only | “Intellectual disabilities or Mental retardation or Learning difficulties or Special needs” | Disability / Special Needs |
| Communication disorder | Not controlled, free text only | “Disabilities or Communication problems or Disorders of communication or Languages disorder or Language impairments” | Disability / Special Needs |
| Language disorder | Not controlled, free text only | “Language impairment or Specific language disorder” | Disability / Special Needs |
| Developmental disorder | Not controlled, free text only | “Developmental disabilities” | Disability / Special Needs |
| Intellectual disability | Not controlled, free text only | “DEVELOPMENTAL disabilities or INTELLECTUAL disabilities or INTELLECTUALLY disabled persons or MENTAL disabilities, People with or MENTAL health or MENTALLY deficient people or MENTALLY deficient persons or MENTALLY disabled persons or MENTALLY handicapped or MENTALLY retarded people or MENTALLY retarded persons or PEOPLE with disabilities or PEOPLE with mental disabilities or PEOPLE with mental illness or RETARDED” | Disability / Special Needs |
| Behavior disorder | Not controlled, free text only | “Behavior problems or Behavior difficulties” | Disability / Special Needs |
| Emotional difficulty | Not controlled, free text only | - | Disability / Special Needs |
| Social difficulty | Not controlled, free text only | - | Disability / Special Needs |
| Cognitive difficulty | Not controlled, free text only | “Cognition or Cognition Disorders or Cognitive ability” | Disability / Special Needs |
| Child | Not controlled, free text only | “Children or Adolescents or Youth or Child or Teenager” | Age Group |
| Teenager | Thesaurus | “ADOLESCENT psychology or ADOLESCENTS or CHILDREN or HIGH school students or PUBERTY or TEEN-agers or TEENS or YOUNG adults (Teenagers) or YOUTH” | Age Group |
| Adolescent | Not controlled, free text only | “Teenagers or Young adults” | Age Group |
| Youth | Thesaurus | “ADOLESCENT psychology or CHILDREN or TEENAGERS or YOUNG adults or YOUNG people or YOUNG persons or YOUNGSTERS” | Age Group |
| Young adult | Thesaurus | “CHILDREN or TEENAGERS or TRANSITIONAL adults or YOUNG people or YOUNG persons or YOUTH” | Age Group |

**Specific Limiters: Language: English – French**

***Érudit***

***For Érudit, the same terms were used with few modifications and applied to the search strategy in French, as it is a French-language database. As this database is formatted differently, only a few limiters were applied in order to support a broader search and include more studies related to various types of disabilities.***

**EQUATION USED:**

**"Programme*" OU "Intervention*"**

**ET**

**"Activité physique*" OU "Sport*" OU "Exercice*"**

**ET**

**"Handicap*" OU "Besoin particulier*" OU "Handicap sensoriel*" OU "Handicap physique*" OU "Trouble physique*" OU "Trouble de l'apprentissage". OU "Trouble de la communication" OU "Trouble du langage" OU "Trouble du développement" OU "Trouble du comportement" OU "Difficultés émotionnelles" OU "Difficulté sociales" OU "Difficulté cognitives".**

**ET**

**"Enfant*" OU "Adolescent*" OU "Jeune adulte*"**

**Specific limiters: *Tous les champs* for each concept; years 2013-2025**
