# Supplementary material for: Effects of group-based physical activity programs on children, adolescents, and young adults with disabilities: A systematic review
Source: PLoS One. 2025 May 23;20(5):e0323707. doi: 10.1371/journal.pone.0323707 (PMC12101651; doi:10.1371/journal.pone.0323707)
Supplement: S2 List — (DOCX) [file pone.0323707.s002.docx]

**S2 List. Specific disability search (Handsearch)**

**Physical Disabilities**

- Paralysis (e.g., paraplegia, quadriplegia)
- Muscular dystrophy
- Cerebral palsy
- Amputation
- Spinal cord injuries

**Sensory Disabilities**

- Blindness or visual impairment
- Deafness or hearing impairment

**Intellectual Disabilities**

- Down syndrome
- Fragile X syndrome
- Fetal alcohol spectrum disorder (FASD)
- Intellectual developmental disorder (IDD) / Intellectual disability

**Developmental Disabilities**

- Autism spectrum disorder (ASD)
- Attention deficit hyperactivity disorder (ADHD)
- Learning disabilities (e.g., dyslexia, dyscalculia, dysgraphia)
- Developmental coordination disorder

**Psychological Disabilities**

- Depression
- Anxiety disorders
- Schizophrenia
- Epilepsy
- Bipolar disorder
- Obsessive-compulsive disorder (OCD)
- Post-traumatic stress disorder (PTSD)
- Eating disorders (e.g., anorexia, bulimia)
