# Supplementary material for: Effects of group-based physical activity programs on children, adolescents, and young adults with disabilities: A systematic review
Source: PLoS One. 2025 May 23;20(5):e0323707. doi: 10.1371/journal.pone.0323707 (PMC12101651; doi:10.1371/journal.pone.0323707)
Supplement: S3 List — (DOCX) [file pone.0323707.s003.docx]

**S3 List. List of the studies included in the review**

1. Angeli, J. M., Peck, M. N., & Schwab, S. M. (2019). Self-perceived scholastic competence, athleticcompetence, and physical appearance are enhanced in children and young adults with physical disabilities following a community-based running program. *Journal of Developmental and Physical Disabilities*, *31*, 707-723.

2. Ansa, O. E. O., Mprah, K. W., Moses, M. O., Owusu, I., & Acheampong, E. (2021). Effect of community-based functional aerobic training on motor performance and quality of life of children with spastic cerebral palsy. *Ethiopian journal of health sciences*, *31*(2).

3. Bahrami, F., Movahedi, A., Marandi, S. M., & Sorensen, C. (2016). The effect of karate techniques training on communication deficit of children with autism spectrum disorders. *Journal of autism and developmental disorders*, *46*, 978-986.

4. Chen, C. C., Ryuh, Y. J., Fang, Q., Lee, Y., & Kim, M. L. (2019). The effects of inclusive soccer program on motor performance and sport skill in young adults with and without intellectual disabilities. *Journal of Developmental and Physical Disabilities*, *31*, 487-499.

5. Chen, C. C., Ryuh, Y., Hardwick, H., Shirley, R., Brinkley, B., Lim, S., ... & Kim, M. L. (2019). Psychological benefits of inclusive soccer program in young adults with and without intellectual disabilities. *Journal of developmental and physical disabilities*, *31*, 847-861.

6. Choi, P. H. N., & Cheung, S. Y. (2016). Effects of an 8-week structured physical activity program on psychosocial behaviors of children with intellectual disabilities. *Adapted Physical Activity Quarterly*, *33*(1), 1-14.

7. Collins, K., & Staples, K. (2017). The role of physical activity in improving physical fitness in children with intellectual and developmental disabilities. *Research in developmental disabilities*, *69*, 49-60.

8. Ekins, C., Wright, J., Schulz, H., Wright, P. R., Owens, D., & Miller, W. (2019). Effects of a drums alive® kids beats intervention on motor skills and behavior in children with intellectual disabilities. *Palaestra*, *33*(2).

9. Hsu, P. J., Yeh, H. L., Tsai, C. L., Chu, C. H., Chen, F. C., & Pan, C. Y. (2021). Effects of a floor hockey intervention on motor proficiency, physical fitness, and adaptive development in youths with mild intellectual disabilities. *International journal of environmental research and public health*, *18*(13), 7059.

10. Kokaridas, D., Demerouti, I., Margariti, P., & Krommidas, C. (2018). The Effect of an Indoor Climbing Program on Improving Handgrip Strength and Traverse Speed of Children With and Without Autism Spectrum Disorder. *Palaestra*, *32*(3).

11. Mohanty, S., Pradhan, B., & Hankey, A. (2019). Yoga practices as an alternative training for physical fitness in children with visual impairment. *Adapted Physical Activity Quarterly*, *36*(4), 431-446.

12. Morales, J., Fukuda, D. H., Garcia, V., Pierantozzi, E., Curto, C., Martínez-Ferrer, J. O., ... & Guerra-Balic, M. (2021). Behavioural improvements in children with autism spectrum disorder after participation in an adapted judo programme followed by deleterious effects during the COVID-19 lockdown. *International Journal of Environmental Research and Public Health*, *18*(16), 8515.

13. Perić, D. B., Milićević‐Marinković, B., & Djurović, D. (2022). The effect of the adapted soccer programme on motor learning and psychosocial behaviour in adolescents with Down syndrome. *Journal of Intellectual Disability Research*, *66*(6), 533-544.

14. Pejčić, A., & Kocić, M. (2020). The impact of sport games exercise programs on the development of specific motor abilities in adolescents with intellectual impairment. *Facta Universitatis, Series: Physical Education and Sport*, (1), 249-261.

15. Phung, J. N., & Goldberg, W. A. (2019). Promoting executive functioning in children with autism spectrum disorder through mixed martial arts training. *Journal of autism and developmental disorders*, *49*, 3669-3684.

16. Pierantozzi, E., Morales, J., Fukuda, D. H., Garcia, V., Gómez, A. M., Guerra-Balic, M., & Carballeira, E. (2022). Effects of a long-term adapted judo program on the health-related physical fitness of children with ASD. *International Journal of Environmental Research and Public Health*, *19*(24), 16731.

17. Radenković, M., Berić, D., & Kocić, M. (2014). The influence of the elements of basketball on the development of motor skills in children with special needs. *Facta Universitatis, Series: Physical Education and Sport*, 123-130.

18. Ryuh, Y., Choi, P., Oh, J., Chen, C. C., & Lee, Y. (2019). Impact of inclusive soccer program on psychosocial development of children with and without intellectual disabilities. *Journal of Developmental and Physical Disabilities*, *31*, 691-705.

19. Stojanović, M., Aleksandrović, M., & Aleksić-Veljković, A. (2018). The effects of exercise program on the balance of young people with intellectual disabilities. *Facta Universitatis, Series: Physical Education and Sport*, *16*(1), 221-228.

20. Xu, C., Yao, M., Kang, M., & Duan, G. (2020). Improving physical fitness of children with intellectual and developmental disabilities through an adapted rhythmic gymnastics program in China. *BioMed Research International*, *2020*.
