# Supplementary material for: Effects of group-based physical activity programs on children, adolescents, and young adults with disabilities: A systematic review
Source: PLoS One. 2025 May 23;20(5):e0323707. doi: 10.1371/journal.pone.0323707 (PMC12101651; doi:10.1371/journal.pone.0323707)
Supplement: S1 Table — (DOCX) [file pone.0323707.s004.docx]

**S1 Table. Inclusion and Exclusion Criteria**

| **Category** | **Inclusion Criteria** | **Exclusion Criteria** |
| --- | --- | --- |
| **Study Period** | - Studies published between 2013 and 2025 | - Studies published before 2013 or ongoing/unpublished studies |
| **Language** | - Published in English or French | - Studies in other languages |
| **Participants** | - Children, adolescents, or young adults (aged 5–24 years) - Diagnosed with a disability (developmental, learning, emotional, physical, intellectual, sensory) | - Participants under 5 years old or over 24 years old - Studies that do not explicitly define the disability or include only typically developing participants - Studies on temporary impairments (e.g., short-term injuries) |
| **Intervention** | - Group-based physical activity (PA) or sports program involving at least 3 participants with disabilities - Supervised by a coach, facilitator, or educator (paid or volunteer) - Conducted in community settings (e.g., gym, park, pool, recreation center) - Minimum duration of 2 weeks - Inclusive programs where participants with and without disabilities engage together (not mandatory) - Group-based PA must involve social   interaction (e.g., team sports, cooperative games) | - Individual or one-to-one interventions (e.g., personal training, paired activities with adults only) - Therapy-based, school-based, or competitive sports programs (requiring a high level of professional oversight) - Home-based or online PA interventions (e.g., home exercise programs, Wii, VR, online workouts) - PA programs lasting less than 2 weeks   Studies where the program involves only  typically developing participants   - Solo PA activities unless conducted in a group setting (e.g., swimming, running, fitness) - Pair sports that limit social interaction (e.g., tennis, badminton; limited to only 2 individuals) - Mixed interventions where PA is secondary (e.g., weight loss programs, multi-component interventions) |
| **Outcomes** | - Reports at least one of the following outcomes:   - Cognitive (e.g., attention, memory) - Affective (e.g., self-esteem, emotional well-being) - Physical (e.g., strength, endurance) - Social (e.g., interaction, communication)   - Reports program characteristics (length, frequency, intensity, setting, trainer qualifications, adaptations) | - Studies that do not assess PA-related effects or include irrelevant outcome measures (e.g., attendance rate, blood pressure, cost-effectiveness, etc.) - Studies with unclear or missing descriptions of interventions and outcomes |
| **Study Design** | - Quasi-experimental studies and randomized controlled trials (RCTs) | - Non-empirical studies (e.g., opinion pieces, conceptual papers) - Studies with no results section (descriptive) - Reviews, meta-analyses, theses, grey literature - Qualitative-only studies (e.g., interviews, case studies with no PA effect measures) |
