# Supplementary material for: Effects of group-based physical activity programs on children, adolescents, and young adults with disabilities: A systematic review
Source: PLoS One. 2025 May 23;20(5):e0323707. doi: 10.1371/journal.pone.0323707 (PMC12101651; doi:10.1371/journal.pone.0323707)
Supplement: S2 Table — (DOCX) [file pone.0323707.s005.docx]

**S2 Table. General description of the retained studies**

| **Reference** | **Participants** | **Study Design** | **Study quality** |
| --- | --- | --- | --- |
| 1.Bahrami, F., et al. (2016). | **Total *N*** = 30  (26 males, 4 females)  **Exercise**  (*n* = 15)  **Control group**  (*n* = 15)  **Disability**: ASD  **Age:**  5-16 years old,  *M* = 9.13 ± 3.27 years | Longitudinal  (randomly assigned) | High risk  (RoB 2) |
| 2. Chen, C. C., et al. (2019) | **Total *N*** = 20  **With ID**  (*n* = 10; 7 males and 3 females)  **Without ID**  (*n* = 10; 10 females)  **Disability:** ID  **Age:**  **ID group:**  *M* = 20.90 ± 1.59 years  **Without ID group:**  *M* = 20.20 ± 1.32 years | Quasi-experimental | Serious risk of bias  (ROBINS-I) |
| 3. Chen, C. C., et al. (2019). | **Total *N*** = 24  W**ith ID**  (*n* = 12; 9 males and 3 females)  **Without ID**  (*n* = 10; 10 females)  **Disability:** ID  **Age:**  **ID group**:  *M* = 20.83 ± 1.47 years  **Without ID group**:  *M* = 20.20 ± 1.32 years | Quasi-experimental | Serious risk of bias  (ROBINS-I) |
| 4. Choi, P. H. N. and S. Y. Cheung (2016). | **Total *N*** = 30  (22 boys, 8 girls)  **Training group**  (*n* = 18)  **Control group**  (*n* = 12)  **Disability:** all had ID + ASD (60% of them)  **Age:**  *M* = 7.39 ± .50 years | Quasi-experimental | Moderate risk of bias  (ROBINS-I) |
| 5.Collins, K. and K. Staples (2017). | **Total *N*** = 35  (25 boys, 10 girls)  **Disability:**  ASD (*n* = 23), DS (*n* = 3), fragile-x (*n* = 2), fetal alcohol spectrum disorder (*n* = 2), developmental delay (*n* = 5)  **Age:**  7-12 years old,  *M* = 9.33 ± 1.82 years | Quasi-experimental | Moderate risk of bias  (ROBINS-I) |
| 6. Angeli, J. M., et al. (2019). | **Total *N*** = 20  (17 males, 3 females)  **Disability:** physical disabilities  **Age:**  7-24 years old,  *M* = 14.2 ± 4.6 years | Quasi-experimental | Moderate risk of bias  (ROBINS-I) |
| 7. Ryuh, Y., et al. (2019). | **Total *N*** = 40  **Inclusive program**  (*n* = 20)  **Segregated program**  (*n* = 20)  *(n* = 10 ID; *n* = 10 without ID per group)  **With ID** (*n* = 20)  **Without ID** (*n* = 20)  **Disability:** ID  **Age:**  **With ID:**  *M* = 10.9 ± 0.6 years  **Without ID:**  *M* = 10.6 years ± 0.7 years | Quasi-experimental | Serious risk of bias  (ROBINS-I) |
| 8. Ansa, O. E. O., et al. (2021). | **Total *N*** = 32  **Disability:** ID (*n =* 8), CP (*n* = 20), DS (*n* = 4)  **Age:**  11-17 years old | Quasi-experimental | Serious risk of biais  (ROBINS-I) |
| 9. Morales, J., et al. (2021). | **Total *N*** = 11  (7 boys, 4 girls)  **Disability:** ASD  **Age:**  9-13 years old,  *M* = 10.17 ± 2.45 years | Longitudinal  (non-random) | Serious risk of biais  (ROBINS-I) |
| 10. Perić, D. B., et al. (2022). | **Total *N*** = 25 males  **Exercise group**  (*n* = 12)  **Control group**  (*n* = 13)  **Disability:** DS  **Age:**  15-17 years old,  *M* = 15.75 years | Experimental | High risk of biais  (RoB 2) |
| 11. Hsu, P.-J., et al. (2021). | **Total *N*** = 54  **Exercise Group** (EG)  (*n* = 27)  **Control Group** (CG)  (*n* = 27)  **Disability:** ID  **Age:**  15-17 years old,  (**EG**) *M* = 16.59 ± 0.56 years  **(CG**) *M* = 16.65 ± 0.63 years | Experimental | High risk of biais  (RoB 2) |
| 12. Xu, C., et al. (2020). | **Total *N*** = 22  (13 boys, 9 girls)  **Experimental group**  (*n* = 12);  ASD (*n* = 4), ID (*n* = 5), and DS *n* = 3)  **Control group**  (*n* = 10);  ASD (*n* = 4), ID (*n* = 3), and DS (*n* = 3)  **Disability:** IDD  **Age:**  **Experimental group:**  *M* = 7.2 years  **Control group:**  *M* = 7.5 years | Quasi-experimental | Moderate risk of bias  (ROBINS-I) |
| 13. Ekins, C., et al. (2019). | **Total *N*** = 15  (11 males, 4 females)  **Intervention group**  (*n* = 9)  **Comparison group**:  (*n* = 5)  **Disability:** ID  **Age:**  *M* = 13.9 ± 2.7 years | Quasi-experimental | Serious risk of bias  (ROBINS-I) |
| 14. Pejčić, A. and M. Kocić (2020). | **Total *N*** = 60  **Experimental group** (EG)  (*n* = 30)  **Control group** (CG):  (*n* = 30)  **Disability:** ID  **Age:**  13-17 years old | Quasi-experimental | Moderate risk of biais  (ROBINS-I) |
| 15. Radenković, M., et al. (2014). | **Total *N*** = 27  **Experimental**  (*n* = 13)  **Control sub-sample**  (*n* =14)  **Disability:** ID  **Age:**  16-19 years old | Longitudinal study  (non-random) | Serious risk of biais  (ROBINS-I) |
| 16. Stojanović, M., et al. (2018). | **Total *N*** = 15  **Disability:** ID  **Age:**  15-20 years old,  *M* = 18.18 ±1.54 years | Quasi-experimental | Serious risk of biais  (ROBINS-I) |
| 17. Kokaridas, D., et al. (2018). | **Total *N*** = 6  **With ASD**  (*n* = 3)  **Without ASD**  (*n* = 3)  **Disability:** ASD  **Age:**  9 years old | Quasi-experimental | Serious risk of biais  (ROBINS-I) |
| 18. Mohanty, S., et al. (2019). | **Total *N*** = 83  **Yoga group** (YG)  (*n* = 41)  **Control group** (CG)  (*n* = 42)  **Disability:** Visual Impairment (VI)  **Age:**  9-16 years old,  **(YG)** *M* = 12.00 ± 2.03 years  **(CG)** *M* = 12.74 ± 2.32 years | Quasi-experimental | Serious risk of biais  (ROBINS-I) |
| 19.Pierantozzi, E.et al. (2022) | **Total *N*** = 40  **Experimental group**  (*n* = 21)  **Control group**  (*n* = 19)  **Disability:** ASD  **Age:**  *M* = 11.07 ±1.73 years | Quasi-experimental | Moderate risk of biais  (ROBINS-I) |
| 20.Phung, J.N et al. (2019) | **Total *N* =** 34  (28 boys)  **Experimental; Mixed martial arts (MMA)**  (*n* = 14)  **Waitlist control**  (*n* = 20)  **Disability:** ASD  **Age:**  *M* = 9.34 ± 1.08 years | Experimental | High risk of biais  (RoB 2) |

*Note.* ID = intellectual disability; IDD = intellectual and developmental disabilities; DCD = developmental coordination disorder; ASD = autistic spectrum disorder; DS = down syndrome; CP= cerebral palsy; *N* = number of participants in the study; *n* = number of participants in the condition; *M* = mean.
