# Supplementary material for: Effects of group-based physical activity programs on children, adolescents, and young adults with disabilities: A systematic review
Source: PLoS One. 2025 May 23;20(5):e0323707. doi: 10.1371/journal.pone.0323707 (PMC12101651; doi:10.1371/journal.pone.0323707)
Supplement: S3 Table — (DOCX) [file pone.0323707.s006.docx]

**S3 Table. Programs’ settings.**

| **Reference** | **Program description** | **Program recommendations** |
| --- | --- | --- |
| 1.Bahrami, F., et al. (2016). | **Program’s name:** N/A  **Length:** 14 weeks – 56 sessions  **Frequency:** 4 times per week  **Intensity:** 30-90 mins (progressive)  **Physical activity:** Kata; Karate techniques (martial art)  **Trainers:** 20 qualified and certified trainers  **Training:** 20h training course assisted by an expert  **Group setting:** divided into 1:1 instruction followed by synchronized group practice  **Additional details:** used motivational techniques  **Fidelity of implementation:** N/A | Additional studies are needed as well as investigations of specific mechanisms demonstrating communication benefits of physical exercise for children with ASD. |
| 2. Chen, C. C., et al. (2019) | **Program’s name:** N/A  **Length:** 15 weeks  **Frequency:** 2 times per week  **Intensity:** 50 mins per session  **Physical activity:** Soccer  **Trainers:** coaches with experience for teaching individuals with ID  **Training:** N/A  **Group setting:** peered participants (ID participant with participant without ID)  **Additional details:** sessions are as follow: warm-up (10 mins), practice (20 mins), soccer games (10 mins), cool-down (10 mins)  **Fidelity of implementation:** N/A | Social support in the inclusive sport environment may be a good platform that provided feeling of enjoyment or interest to participants with ID and moved their sport motivation from extrinsically toward intrinsically.  Future studies will be needed to understand whether exercise can lead to sustained psychological benefits in this population.  Future studies should also include other physiological measures, such as EEG and enjoyment scale, to identify their potential relationship between physical activity, cognitive performance and affective response. |
| 3. Chen, C. C., et al. (2019). | **Program’s name:** N/A  **Length:** 15 weeks  **Frequency:** 2 times per week  **Intensity:** 50 mins per session  **Physical activity:** Soccer  **Trainers:** soccer coaches with experience for teaching individuals with ID  **Training:** N/A  **Group setting:** peered participants (ID participant with participant without ID)  **Additional details:** sessions are as follow: warm-up exercises, practice, soccer games, cool-down  **Fidelity of implementation:** N/A | Future studies may consider the past sport experience or accomplishment as the covariant into data analysis.  Further investigations are still needed to study with larger sample size, more reliable measures as well as participants’ psychological attributes.  Findings can encourage coaches, school teachers, and community leaders to implement the inclusive sport programs in their respective areas and may exert a positive influence on attitudes and awareness, education and policy changes to ensure greater sport opportunities for individuals with ID. |
| 4. Choi, P. H. N. and S. Y. Cheung (2016). | **Program’s name:** N/A  **Length:** 8 weeks  **Frequency:** 3 times per week (24 sessions)  **Intensity:** 60 mins  **Physical activity:** Physical activity stations  **Trainers:** 6 instructors  **Training:** undergraduate students who had experience in providing activities to children with ID and had been involved in the pilot study  **Group setting:** 3 groups of 6 participants  **Additional details:** each station lasted for 15 mins  **Fidelity of implementation:** N/A | The results of the study suggest that the after-school time frame can be used to supplement the teaching from special-school teachers.  Currently, after-school programs rarely exist.  Future longitudinal studies are needed to explore the long-term benefits of PA participation and the effectiveness of short PA programs in motivating and sustaining PA participation in children with ID.  Further studies have larger sample sizes and incorporate more schools to examine the interaction of the intervention program with the environment on effecting change in children. |
| 5.Collins, K. and K. Staples (2017). | **Program’s name:** N/A  **Length:** 10 weeks  **Frequency:** once a week  **Intensity:** 90 mins  **Physical activity:** Development of fundamental movements skills, sports, physical activity stations related to sport  **Trainers:** coaches were undergraduate students in kinesiology, education, and social work  **Training:** N/A  **Group setting:** divided in 4 teams  **Additional details:** 2 different sports each week  **Fidelity of implementation:** N/A | Programming needs to provide an opportunity to participate yet find a balance between improving fitness and creating a fun environment where the child is more apt to want to return for the next session.  The programs available must be feasible and realistic for families of children with IDD to commit to.  While school is where students spend the majority of their day, opportunities to be active also need to extend beyond school hours.  Future research is needed to examine how well these physical fitness improvements are maintained and determine to what extent improved levels of fitness translate into increased participation in PA and trajectories of health, including obesity. |
| 6. Angeli, J. M., et al. (2019). | **Program’s name:** N/A  **Length:** 10 weeks  **Frequency:** 2 times per week  **Intensity:** 60 mins  **Physical activity:** Community running  **Trainers:** volunteer coaches and lead physical therapist  **Training:** N/A  **Group setting:** group training sessions were held on non-consecutive days of the week, participants engaged in independent easy walking, defined as the ability to maintain easy conversation, twice per week when not participating in group running practices or days of rest  **Additional details:** running practices followed a time-based progression, all running practices also included periods of warm-up and cool-down, 5–10 min in duration  **Fidelity of implementation:** N/A | These results motivate continued administration of community-based fitness programming as a compliment to traditional physical therapy interventions for children and young adults with physical disabilities and a longitudinal and systematic examination of its distal effects. |
| 7. Ryuh, Y., et al. (2019). | **Program’s name:** N/A  **Length:** 4 weeks  **Frequency:** 5 times per week (total 20 days)  **Intensity:** 90 mins  **Physical activity:** Soccer program  **Trainers:** N/A  **Training:** N/A  **Group setting:** one-on-one peer buddy with participant with no ID  **Additional details:** 10 min of warm up activity with non-competitive physical activity, 30 min of practice with a peer buddy, a 10 min recess period, 30 min of soccer game activity, and 10 min of cool-down  **Fidelity of implementation:** N/A | Encourage positive contact experience within sporting environments to improve inclusivity.  Conduct subsequent studies to examine the impact of sporting environments on psychosocial development and inclusion. |
| 8. Ansa, O. E. O., et al. (2021). | **Program’s name:** CBFAE  **Length:** 8 weeks  **Frequency:** 4 times per week  **Intensity:** 50 mins  **Physical activity:** A functional aerobic exercise  **Trainers:** supervised by professional exercise physiologists  **Training:** N/A  **Group setting:** Exercise sessions were conducted in group station of exercises  **Additional details:** participants warmed-up and cooled-down for ten minutes respectively prior to and at the completion of each session, participants trained at each station for 5–6 minutes totaling 30 minutes, 2 - 3 minutes of rest amounting to 10 minutes and then moved to the next station following completion of each exercise  **Fidelity of implementation:** N/A | Clinicians and exercise therapists should essentially incorporate CBFAE training and activities into the management of children with CP in general and spastic CP specifically for improved mobility and functional performances. |
| 9. Morales, J., et al. (2021). | **Program’s name:** N/A  **Length:** 8 weeks  **Frequency:** once a week  **Intensity:** 75 mins  **Physical activity:** Judo  **Trainers:** 2 judo teachers with degrees in pedagogy and sports sciences and 7th and 6th-degree black belts, respectively, led each session, and at least four volunteer judo instructors were present to lend support  **Training:** N/A  **Group setting:** N/A  **Additional details:** the sessions were divided into a warm-up, main exercise, and cool-down activities  **Fidelity of implementation:** N/A | The long-term effects of these interventions also need to be explored, while additional aspects of behavior, including issues connected to motor skills, should be examined. |
| 10. Perić, D. B., et al. (2022). | **Program’s name:** N/A  **Length:** 16 weeks  **Frequency:** 2 times per week  **Intensity:** 60 mins  **Physical activity:** Soccer  **Trainers:** 3 qualified soccer coaches who were certified in the FIFA Programme (level B), led the sessions with a specialist in adapted physical activity who assisted the participants.  **Training:** N/A  **Group setting:** cooperation of two and three players during exercises  **Additional details:** each session consisted of a 10 min warm-up (running and shuttle run with ball), 45 min of training and a 5 min of cool-down (stretching exercises)  **Fidelity of implementation:** N/A | Future studies should investigate whether 16-week intervention is a long enough period to provoke more serious adaptation in motor learning.  Implement programs in inclusive conditions because they encourage the development of psychosocial skills through team tasks. |
| 11. Hsu, P.-J., et al. (2021). | **Program’s name:** N/A  **Length:** 12 weeks  **Frequency:** 3 times per week  **Intensity:** 90 mins  **Physical activity:** Floor hockey exercise  **Trainers:** primary coach and two research assistants  **Training:** the primary investigator trained one primary coach and two research assistants before delivering any lessons to ensure that they were aware of the lesson focus and required drills so that each lesson would be delivered according to the specified protocol. The primary coach had been an elite national baseball player for 1 year and had 6 years of experience teaching floor hockey exercise to youths with IDs. The primary coach and research assistants majored in physical education, adapted physical education, or special education. They had teaching experience with individuals with IDs  **Group setting:** N/A  **Additional details:** (a) warm-up activities (15 min), (b) floor hockey training (45 min), (c) games and physical fitness training (15 min), and (d) cool-down activities (15 min)  **Fidelity of implementation:** N/A | Future studies should include larger samples and consider various ID severities, school settings, PA levels, and family socioeconomic status of youths with IDs.  Investigating the possible sustained intervention effects in improving outcome variables may yield useful findings. |
| 12. Xu, C., et al. (2020). | **Program’s name:** ARG  **Length:** 16 weeks  **Frequency:** 3 times per week  **Intensity:** 50 mins  **Physical activity:** Gymnastic  **Trainers:** teachers majoring in gymnastics, instructor with expertise in working with disabilities  **Training:** N/A  **Group setting:** N/A  **Additional details:** the contents of the ARG program were separated into three categories: controlled movements, uncontrolled movements, and locomotor skills, warm-up (5 mins), core activities (35 mins) and cool-down (5 mins)  **Fidelity of implementation:** N/A | Use music to satisfy children’s attention needs. Previous research has shown that music or rhythm can help increase the attention of IDD children.  Future research could be needed to examine how well these physical fitness improvements are maintained. |
| 13. Ekins, C., et al. (2019). | **Program’s name:** Drums Alive Kids Beats®  **Length:** 7 weeks  **Frequency:** 2 times per week  **Intensity:** N/A  **Physical activity:** Group drumming  **Trainers:** physical education teachers  **Training:** N/A  **Group setting:** N/A  **Additional details:** warm up, main phase and cool-down also included strength based, team-oriented and relaxing exercises  **Fidelity of implementation:** N/A | A safe and successful Drums Alive® Kids Beats program and its utility in the diverse ability population depends on the instructor’s application of sound instructional principles and practices as well as understanding the desired learning objectives used to develop the research protocols and subsequent positive results:  Safety first.  Know you audience.  Be prepared for medical emergencies.  Use appropriate equipment.  Provide an enriched environment.  Adapt music and tempo. |
| 14. Pejčić, A. and M. Kocić (2020). | **Program’s name:** N/A  **Length:** 12 weeks  **Frequency:** 4 times per week  **Intensity:** 30 mins  **Physical activity:** Sport games (football and basketball)  **Trainers:** N/A  **Training:** N/A  **Group setting:** N/A  **Additional details:** during that time, the participants mastered the technical elements for football (leading the ball, shooting at the goal and passing the ball) and basketball (leading the ball, catching the ball, passing the ball and shooting at the basket) during the main part of the lesson. During each lesson, the participants performed two or more elements by ten repetitions for a warm-up and for raising the muscle tonus that should be activated during the main part of the lesson. The last five minutes were reserved for muscle relaxation and body preparation  **Fidelity of implementation:** N/A | It is in the adolescent period, when the body is ready to endure the biggest changes during its development, the advantages of this program should be used in the best possible way. |
| 15. Radenković, M., et al. (2014). | **Program’s name:** N/A  **Length:** 4 weeks  **Frequency:** 2 times per week  **Intensity:** N/A  **Physical activity:** Basketball  **Trainers:** physical education teacher  **Training:** N/A  **Group setting:** N/A  **Additional details:** warm-up, the main part consisted of the basketball program and the final part consisted of class relaxation exercise. week 1 = Dribbling, weeks 2-3 = passing, week 4 = shooting  **Fidelity of implementation:** N/A | This study can be used as a pilot project for some larger and more extensive research of a longer duration, in order for the results to be more valid and more useful for pedagogical theory and practice. |
| 16. Stojanović, M., et al. (2018). | **Program’s name:** N/A  **Length:** 12 weeks  **Frequency:** 2 times per week  **Intensity:** 45 mins  **Physical activity:** Balls and polygons  **Trainers:** physical education teacher  **Training:** N/A  **Group setting:** N/A  **Additional details:** warm-up (running), main part (ball games), final part (relaxation and breathing)  **Fidelity of implementation:** N/A | Tasks in the form of polygons are extremely interesting for the studied population, so it is further recommended that this kind of work should be applied more often. |
| 17. Kokaridas, D., et al. (2018). | **Program’s name:** N/A  **Length:** 12 weeks  **Frequency:** 2 times per week  **Intensity:** 40 mins  **Physical activity:** Climbing  **Trainers:** N/A  **Training:** N/A  **Group setting:** every child began at a station with the two of the three children in each group asked to climb  **Additional details:** 5 min warm-up period, including balance, coordination, and trunk exercises performed to promote fun and socialization, 30 min of indoor climbing. Each climbing session was structured around three circle line “stations” and there was resting period between stations. Cool-down period at the end (5 mins)  **Fidelity of implementation:** N/A | Future research with larger samples of children with and without ASD, and different levels of functioning for children with developmental disabilities are needed to further ascertain the positive effect of climbing on improving skills that this leisure activity cultivates. |
| 18. Mohanty, S., et al. (2019). | **Program’s name:** N/A  **Length:** 16 weeks  **Frequency:** 5 times per week  **Intensity:** 60 mins  **Physical activity:** Yoga  **Trainers:** certified yoga trainers with experience of teaching yoga to children with VI  **Training:** N/A  **Group setting:** were divided into 4 subgroups of 10 or 11  **Additional details:** (a) breathing practices, (b) loosening practices, (c) yoga asanas (postures), (d) pranayama (regulation of breath), and (e) relaxation  **Fidelity of implementation:** N/A | Further studies may assess yoga for children with VI for neuromuscular dynamics, psychosocial stresses, clinical applications, other sensory functions, and gender differences.  Recommending yoga as an alternative to physical activity program for those with VI. |
| 19.Pierantozzi, E.et al. (2022) | **Program’s name:** N/A  **Length:** 6 months  **Frequency:** once a week  **Intensity:** 90 mins  **Physical activity:** Judo  **Trainers:** 2 judo teachers with 7th and 6th DAN levels led the sessions and 4 volunteer judo instructors  **Training:** N/A  **Group setting:** N/A  **Additional details:** warm-up, main exercise and cool-down  **Fidelity of implementation:** N/A | Further studies should investigate the dose-response relationships of judo training with the aim of reducing cardiovascular risk and improving fitness in this population. |
| 20.Phung, J.N et al. (2019) | **Program’s name: N/A**  **Length:** 13 weeks  **Frequency:** 2 times per week  **Intensity:** 45 mins  **Physical activity:** Mixed martial arts (MMA)  **Trainers:** team of senior martial arts instructors  **Training: N/A**  **Group setting:** 12 children with ASD per class  **Additional details:** 5 mins (bow-in; sit in meditation), 15 mins (warm-up; jogging, stretching), 20 mins (main activity; MMA), 5 mins (cool-down; social game). Curriculum per weeks; 1-4 weeks = Sticking, 5 to 8 weeks = Glove drills, 9 to 13 weeks = combination  **Fidelity of implementation:** Implementation fidelity adherence ranged from 73 to 100% | Future research should examine which specific components of cognitive regulation, if any, can be feasibly targeted by martial arts training.  Future research also is needed to compare outcomes for specific martial arts styles (e.g., judo, karate, kickboxing, etc.)  Longitudinal follow up of study participants would be useful to see whether the significant executive functioning benefits endure. |

*Note.* ID = intellectual disability; IDD = intellectual and developmental disabilities; DCD = developmental coordination disorder; ASD = autistic spectrum disorder; DS = down syndrome; CP= cerebral palsy.
