# Supplementary material for: Effects of group-based physical activity programs on children, adolescents, and young adults with disabilities: A systematic review
Source: PLoS One. 2025 May 23;20(5):e0323707. doi: 10.1371/journal.pone.0323707 (PMC12101651; doi:10.1371/journal.pone.0323707)
Supplement: S4 Table — (DOCX) [file pone.0323707.s007.docx]

**S4 Table. Adaptation details.**

| **Reference** | **Adaptation details** |
| --- | --- |
| 1.Bahrami, F., et al. (2016). | N/A |
| 2. Chen, C. C., et al. (2019) | Program was indoor, with one specific training per week (e.g., ball control week 1-3). |
| 3. Chen, C. C., et al. (2019). | N/A |
| 4. Choi, P. H. N. and S. Y. Cheung (2016). | Pilot studies were done before the intervention to choose the best physical activity stations. |
| 5.Collins, K. and K. Staples (2017). | Coach to child ratio was 1:3. |
| 6. Angeli, J. M., et al. (2019). | The initial 5 weeks of the training program were designed to familiarize participants with the routine, participants were able to select their desired training intervals. While running practices were adapted when necessary to meet unique needs and abilities. |
| 7. Ryuh, Y., et al. (2019). | Disability awareness education was used for typically developing children, usage of the word disability was strictly prohibited to facilitate a cooperative environment and reduce the possibility for any child to develop prejudice. All children attached a removable nametag on the backside of their team jersey which encouraged them to memorize and call out the name of their partners. |
| 8. Ansa, O. E. O., et al. (2021) | During the first day of training, participants were made to familiarize themselves with the equipment, and appropriate intensity of exercise was determined. |
| 9. Morales, J., et al. (2021). | The program was performed in a large and well-ventilated space suitable for judo practice, such that the safety of the participants was maintained. |
| 10. Perić, D. B., et al. (2022). | N/A |
| 11. Hsu, P.-J., et al. (2021). | Program was adapted to individual needs (e.g., different learning pace and ability, execution time), being safe, easy to learn, fun, capable of reaching moderate- to vigorous-intensity PA, some additional practice activities were developed to help participants generalize their skills. In addition, for competitions, emphasis was placed on the rules not being changed to suit the athletes’ special needs. However, the primary coach was permitted to modify the training activities to accommodate each participant’s special needs or adapt sport equipment to assist participants in achieving success. |
| 12. Xu, C., et al. (2020). | Pre-experiment was conducted before the program. |
| 13. Ekins, C., et al. (2019). | The Drums Alive® Kids Beats intervention adopted a holistic approach, based on the children´s needs. |
| 14. Pejčić, A. and M. Kocić (2020). | N/A |
| 15. Radenković, M., et al. (2014). | N/A |
| 16. Stojanović, M., et al. (2018). | The main part of the class content is always selected based on the current mood and emotional state of the students. Different ball games were used during these classes (with tennis balls, volleyball balls, basketballs) to avoid monotony. |
| 17. Kokaridas, D., et al. (2018). | N/A |
| 18. Mohanty, S., et al. (2019). | Audio cassettes with detailed instructions of all practices were provided. |
| 19.Pierantozzi, E.et al. (2022) | The judo sessions were held in an ample, well-ventilated space suitable for athletic activity in general and judo in particular, so the safety of the participants was guaranteed. Each participant was allowed to progress at his or her own pace. One on one support was also given. |
| 20.Phung, J.N et al. (2019) | Children were reminded to use their breathing techniques to help regulate their emotions if they became frustrated or overwhelmed throughout the class. |
