# Supplementary material for: Effects of group-based physical activity programs on children, adolescents, and young adults with disabilities: A systematic review
Source: PLoS One. 2025 May 23;20(5):e0323707. doi: 10.1371/journal.pone.0323707 (PMC12101651; doi:10.1371/journal.pone.0323707)
Supplement: S5 Table — (DOCX) [file pone.0323707.s008.docx]

**S5 Table. Assessments and outcomes of the included programs.**

| **Reference** | **Variables** | **Assessment(s)** | **Outcomes** |
| --- | --- | --- | --- |
| 1.Bahrami, F., et al. (2016). | **DV:** Communication  **IV:** Karate techniques  **CV:** Control group | Communication subscale of Gilliam Autism -  Rating Scale-Second Edition (GARS-2; Gilliam 2006) to assess changes in the severity of communication deficits. | Karate training improved communication deficit of children diagnosed with ASD.  Findings are consistent with findings that physical exercise is beneficial for individuals with neurological and psychiatric conditions, across age and diagnostic categories and for a variety of symptoms and areas of adaptive functioning.  After 30 days of no practice, communication deficit in the exercise group remained significantly decreased compared to post-intervention time. |
| 2. Chen, C. C., et al. (2019) | **DV:** Sport motivation, heart rate, executive function  **IV:** Soccer program  **CV:** Participant without ID | Heart Rate - based on the formula.  provided by Tanaka et al. (2001) (i.e., 208–0.7Xage).  Sport motivation - Adaptation of the Sport Motivation Scale guided by Self-Determination Theory (SDT).  Executive functions - Corsi Block Tapping Test (assess visuo-spatial working memory span) / Eriksen Flanker Test (assess select attention to suppress responses that are inappropriate in a particular context). | The soccer program has psychological benefits on individuals with ID in addition to laboratory settings.  Adults with ID had lower motivation to exercise than their typical peers.  Their self-determined functioning to exercise was improved after the inclusive soccer program although the statistically significant level was not achieved.  Participants with ID only improved their response accuracy but reaction time in congruent and incongruent conditions. |
| 3. Chen, C. C., et al. (2019). | **DV:** Perceive Exertion, manual dexterity, soccer skill performance  **IV:** Soccer program  **CV:** Participant without ID | Heart Rate - based on the formula.  provided by Tanaka et al. (2001) (i.e., 208–0.7Xage).  Rate of Perceive Exertion - The Children’s OMNI walk/run Scale was used to rate  perceived exertion.  Purdue Pegboard Test - The Purdue Pegboard Test was used to evaluate manual dexterity.  6 Min Walk Test – evaluate capacity and mobility.  Special Olympics Soccer Skill Test - The test comprised of dribbling, passing and shooting. | Findings indicated that this inclusive soccer program resulted in a moderate exercise intensity for participants with ID.  Participants with ID reported higher scores in perceive exertion scale compared to their typical partners.  After training, participants with ID had significantly improved in manual dexterity.  Although no significant level was approached in young adults with ID, large effect size in soccer skills was still observed. |
| 4. Choi, P. H. N. and S. Y. Cheung (2016). | **DV:** Psychosocial Behaviors (self-control skills, social interaction)  **IV:** Structured physical activity  **CV:** Control group | Systematic Observation - Behaviors in the training context were recorded on the Checklist for Pupil Evaluation (Wright & Sugden, 1999).  The researcher and an expert on special education served as observers of the assessment. | Training group participants demonstrated a gradual improvement in emotional self-control after the PA program in both the training context and the classroom context.  The current study also supports the claim that early intervention should be implemented to minimize psychosocial-development delays in children with ID.  Benefit the psychosocial development of children with ID. |
| 5.Collins, K. and K. Staples (2017). | **DV:** Physical fitness (body composition, aerobic functioning and musculoskeletal functioning)  **IV:** Physical activity program  **CV:** N/A | The Brockport Physical Fitness Test - body mass  index (body composition), 20 m PACER run (aerobic functioning), modified curl up and isometric push up (muscular strength and  endurance) and modified sit and reach (flexibility). | All participants improved on at least one component of health-related fitness (body composition, aerobic functioning and musculoskeletal functioning).  Structured PA programming and improved motor competence can lead to improved levels of physical fitness among children with IDD. |
| 6.Angeli, J. M., et al. (2019). | **DV:** Self-perception  **IV:** Running community-based program  **CV:** N/A | The Self-Perception Profile for Children (SPPC Harter 2012) – evaluate changes in self-concept. | Results suggest that a community-based model of physical activity can positively influence self-concept in multiple domains, with significant improvements noted in the domains of scholastic competence, athletic competence, and physical appearance. |
| 7. Ryuh, Y., et al. (2019). | **DV:** Social age, social distance, withdrawn behavior  **IV:** Inclusive Soccer program (participants are mixed)  **CV:** Segregated soccer program | Social Age - assessed and evaluated by the Korean version of the social maturity scale (Lee et al. 2014).  Social Distance Scale (SDS) – evaluate social distance of children toward peers with ID based on ‘Disability-Acceptance Scale: Behavioral  Intention’ (김성애, 정대영,, and 박희찬 1997).  Withdrawn Behavior Checklist (WBC) - assessing the behavior of children with ID, and was divided into three categories: friendships-related, communication-related, and self-esteem-related withdrawn behavior. | The effectiveness of an inclusive soccer program has provided the evidence that physical activity program which integrates children with and without ID can reduce the withdrawn behavior and social distance of both typically developing children as well as children with ID.  Administering a high quality inclusive soccer program with positive contact experience may be a significant contributor for achieving successful outcome.  The result found that the training (education awareness) was significantly beneficial to improve the self-reported attitude of children toward people with disabilities.  The peer buddy system within the program possibly facilitated perceptions of similarity, or equal status, within each pair of children with and without ID.  An inclusive soccer program with embedded one-time disability awareness education and peer buddy system confirmed its effectiveness on the psychosocial development of children to achieve true inclusion in sporting environment. |
| 8. Ansa, O. E. O., et al. (2021). | **DV:** Gross motor function, walking distance, quality of life  **IV:** Community-based functional aerobic exercise  **CV:** N/A | Gross Motor Function scale (GMFM) - evaluate gross motor function of the participants.  Walking Distance - the 30 Seconds Walk  Test (30SWT) assesses coordination and exercise capacity associated with walking ability over a given distance with a given time.  Quality of life – Cerebral palsy quality of life questionnaires (CPQoL). | Improved gross motor functions, walking distance and quality of life of CP children.  These findings substantiate the submission on exercise regimen being the most effective interventions for CP management. |
| 9. Morales, J., et al. (2021). | **DV:** Repetitive behaviours, social interaction, social communication, emotional responses, cognitive style and maladaptive speech  **IV:** Judo  **CV:** Control period and Confinement period | Gilliam Autism Rating Scale-Third Edition (GARS-3) - assess changes in the severity of ASD behaviours. | Confirms the positive effects of 8-week adapted judo training in repetitive behaviours, social interaction, social communication and emotional responses subscales in children with ASD.  Confirm prior research examining the use of combat sports or martial arts to improve certain executive and psychosocial behaviours that also influence the quality of life of children with ASD.  Support and confirm previous findings showing that participation in sport can improve the social abilities of children with ASD and help improve engagement in social interactions. |
| 10. Perić, D. B., et al. (2022). | **DV:** Motor learning and some psychosocial characteristics  **IV:** Soccer program  **CV:** Control group | Psychosocial variables assessment – in cooperation with the psychologists of the specialisedinstitution CEC, the authors compiled a list of items based on previously used instruments whose metrics are available in the literature (Achenbach et al. 2005; Lecavalier and Butter 2010) ; aggression, attention disorders, anxiety and depression and social problems.  Soccer skills assessment - Motor task of the level 1 (the easiest task): straight dribbling (shuttle run between two cones 10m away with the ball control); Motor task of level 2 (moderately difﬁcult task): dribbling with the ball stopping (the participant stops the ball three times at the marked place during the dribble between cones); Motor task of level 3 (the most difﬁcult task): slalom dribbling between ﬁve cones with goalkicking. | Results suggest that adapted soccer training can decrease aggression, anxiety and depression levels, and improve attention, social behaviour and simple motor skills in adolescents with Down syndrome. |
| 11. Hsu, P.-J., et al. (2021). | **DV:** Motor proficiency, physical fitness, and adaptive development  **IV:** Floor hockey exercise group  **CV:** Control group | Motor Proficiency - assessed using the standardized assessment of the BOT-2; measure fine and gross motor proficiency.  Physical Fitness – The Brockport Physical Fitness Test (BPFT); assess the physical fitness components of the participants and included: (a) the 20 m Progressive Aerobic Cardiovascular Endurance Run (PACER) test, (b) a modified curl-up test, (c) an isometric push-up test, and (d) the back-saver sit-and-reach test.  Adaptive Development - ABAS-II; measures nine skill areas and yields four composite scores for the following skill categories: conceptual (e.g., telling teachers, friends, or others your favorite activities; reading important documents, for example, registration notice or leave policy),  social (e.g., inviting and letting others play games or other interested activities, saying  please when asking for a favor), practical (e.g., choosing appropriate clothing based on  weather, asking for help when someone is hurt), and general adaptive skills. | Floor hockey training program significantly increased scores for most indicators of motor proficiency (i.e., manual coordination, body coordination, and total motor composite scores), physical fitness (i.e., cardiovascular fitness, abdominal strength, and endurance scores), and adaptive development (i.e., conceptual and social composite). |
| 12. Xu, C., et al. (2020). | **DV:** Physical fitness  **IV:** Adapted Rhythmic Gymnastics Program (ARG)  **CV:** Control group | Physical fitness – Brockport Physical Fitness Test; parameters in BPFT: body mass index (BMI), 10 m PACER run, curl-ups, dumbbell press, trunk lift, standing long jump, and sit-and-reach test. | Significant improvements were reported for most of the physical fitness parameters, such as muscle strength, aerobic capacity, and explosive strength. |
| 13. Ekins, C., et al. (2019). | **DV:** Effects on physical performance, behavior, cognitive, social and practical competencies  **IV:** Drums Alive® Kids Beats  **CV:** Comparison group | Behavior Questionnaire for Developmental Disabilities (VFL-L) - the VFL-L was used to evaluate the effects on the children’s behavior and emotions.  The Developmental Behavior Checklist (DBC-M) - instrument to monitor children’s behavior during the intervention phase.  The Heidelberg Competency Inventory (HKI) -  assess the cognitive, social and practical competencies of the children.  German Motor Skill Test (DMT) - assess the effects on the participant’s motor skills. | Intervention group showed significant improvement in aerobic performance, coordination and strength. |
| 14. Pejčić, A. and M. Kocić (2020). | **DV:** Motor skills  **IV:** Special exercise program  **CV:** Control group | Motor skills - test battery (Guidetti,Franciosi, Emerenziani, Gallotta, & Baldari, 2009; Stanišić et al., 2012a); four elements: running the ball (SMRB in points), catching the ball (SMCB in points), passing the ball (SMAB in points), and shooting at the basket (SMSB in points). | The twelve-week training program led to statistically significant high changes in specific motor skills in all the variables related to football, as well as in the variables related to basketball, especially in ball control activities.  There was a statistically significant difference in the benefits for the EG compared to the CG.  The results of the current study indicate that a specific exercise program conducted as part of a 12-week training with specifically dosed sports activities has significantly contributed to the development of specific motor skills in the games of football and basketball. |
| 15. Radenković, M., et al. (2014). | **DV:** Motor abilities  **IV:** Basketball  **CV:** Control group | Motor abilities - instruments from the textbook "Anthropological basics of basketball" (Jovanović - Golubović, & Jovanović, 2003; the 20-meter run with a high start, hand tapping, the standing jump, push-ups, depth reach, torso lifts from a bench, throwing dart; | Basketball technique have a statistically significant impact on the development of motor skills. |
| 16. Stojanović, M., et al. (2018). | **DV:** Balance  **IV:** Exercise program  **CV:** N/A | Balance – Bruininks Oseretsky Test of Motor Proficiency, Second Edition (BOT-2, a battery of tests for assessing basic and fine motor skills development). | Significant increase in balance after the program. |
| 17. Kokaridas, D., et al. (2018). | **DV:** Handgrip strength and traverse speed  **IV:** Indoor climbing program  **CV:** Children without disabilities | Handgrip strength – Strength (kg) of dominant hand was measured using the portable Takei Handgrip Test Dynamometer (TKK 5101,  Takei Scientific Instruments Co, Ltd, Tokyo, Japan).  Traverse speed – 5m traverse route timed in seconds. | Improvement for the two skills (handgrip strength and traverse speed) measured and may indicate climbing as a useful recreation tool to improve physical skills of children with ASD.  No statistically significant results within or between groups were noted in handgrip strength though, due to the small sample of the study. However, when all children were grouped into a sample of six participants, statistically significant scores emerged following climbing intervention. |
| 18. Mohanty, S., et al. (2019). | **DV:** Physical fitness (static strength); (flexibility); (trunk strength and endurance); fine motor coordination and speed of limb movement)  **IV:** Yoga intervention  **CV:** Control group | Physical fitness - Eurofit Test Battery: (a) handgrip strength (static strength); (b) sit and reach (flexibility); (c) sit-ups (trunk strength and endurance); (d) plate tapping (fine motor coordination and speed of limb movement), to which was added; and (e) peak expiratory flow rate (PEFR; lung function). | Significant beneficial effects on all the parameters of physical fitness (muscle strength, muscle endurance, flexibility, coordination, and lung function) compared with the control group. |
| 19.Pierantozzi, E.et al. (2022) | **DV:** Cardio-metabolic health and cardiorespiratory fitness  **IV:** Judo program  **CV:** Control group | Cardio-metabolic health and cardiorespiratory fitness - ALPHA-fitness battery; (1) the 20 m Shuttle Run Test to assess cardiorespiratory fitness; (2) the handgrip strength test; (3) the standing long jump tests to assess musculoskeletal fitness; (4) BMI; and (5) waist circumference. | An adapted judo program for children with ASD can improve the cardio-metabolic health and cardiorespiratory fitness of its participants. |
| 20.Phung, J.N et al. (2019) | **DV:** Executive functions: behavioral inhibition, working memory, and cognitive flexibility  **IV:** Mixed martial arts (MMA)  **CV:** Waitlist control | Executive functions - the Lifetime Social Communication Questionnaire (SCQ; Rutter et al. 2003) is 40-item parent report tool used by clinicians to quickly screen for ASD;  the Autism Diagnostic Observation Schedule-Second Edition (ADOS-2) is a standardized, semi-structured assessment of ASD;  the Wechsler Abbreviated Scale of Intelligence-Second Edition (WASI-II; Wechsler 2011) is a brief measure of intelligence; the Hearts & Flowers test is a computerized task that directly measures three core EFs: behavioral inhibition, working memory, and cognitive flexibility (Davidson et al. 2006; Diamond et al. 2007);  the Behavior Rating Inventory of Executive Function (BRIEF-2) is an 86-item parent rating scale designed to assess executive functioning and self-regulation in children and adolescents. | Martial arts training challenged and strengthened the behavior and emotion regulation domains of executive functions such as behavioral inhibition and working memory. |

*Note.* DV = dependant variable; IV = independent variable; CV = control variable; ID = intellectual disability; IDD = intellectual and developmental disabilities; DCD = developmental coordination disorder; ASD = autistic spectrum disorder; DS = down syndrome; CP= cerebral palsy
