# Supplementary material for: Effects of group-based physical activity programs on children, adolescents, and young adults with disabilities: A systematic review
Source: PLoS One. 2025 May 23;20(5):e0323707. doi: 10.1371/journal.pone.0323707 (PMC12101651; doi:10.1371/journal.pone.0323707)
Supplement: S6 Table — (DOCX) [file pone.0323707.s009.docx]

**S6 Table. Results of risk of bias across the studies.**

**ROBINS-I assessment**

| First author & year published | Bias due to confounding | Bias in selection of participants into the study | Bias in classification of interventions | Bias due to deviations from intended intervention | Bias due to missing data | Bias in measurement of outcomes | Bias in selection of  reported result | Overall Risk |
| --- | --- | --- | --- | --- | --- | --- | --- | --- |
| Angeli, 2019 | Moderate risk | Low risk | Low risk | Low risk | Low risk | Moderate risk | Low risk | Moderate risk |
| Ansa, 2021 | Serious risk | Moderate risk | Low risk | Low risk | Low risk | Moderate risk | Low risk | Serious risk |
| Chen, 2019(a) | Serious risk | Low risk | Low risk | Low risk | Low risk | Moderate risk | Low risk | Serious risk |
| Chen, 2019(b) | Serious risk | Low risk | Low risk | Low risk | Low risk | Moderate risk | Low risk | Serious risk |
| Choi, 2016 | Moderate risk | Low risk | Low risk | Low risk | Low risk | Moderate risk | Low risk | Moderate risk |
| Collins, 2017 | Moderate risk | Low risk | Low risk | Low risk | Low risk | Moderate risk | Low risk | Moderate risk |
| Ekins, 2019 | Serious risk | Low risk | Low risk | Low risk | Low risk | Moderate risk | Low risk | Serious risk |
| Kokaridas, 2018 | Serious risk | Low risk | Low risk | Low risk | Low risk | Moderate risk | Low risk | Serious risk |
| Morales, 2021 | Serious risk | Low risk | Low risk | Low risk | Low risk | Moderate risk | Low risk | Serious risk |
| Pejčić, 2020 | Moderate risk | Low risk | Low risk | Low risk | Low risk | Moderate risk | Low risk | Moderate risk |
| Pierantozzi, 2022 | Moderate risk | Low risk | Low risk | Low risk | Low risk | Moderate risk | Low risk | Moderate risk |
| Radenković, 2014 | Serious risk | Low risk | Low risk | Low risk | Low risk | Moderate risk | Low risk | Serious risk |
| Ryuh, 2019 | Serious risk | Low risk | Low risk | Low risk | Low risk | Moderate risk | Low risk | Serious risk |
| Stojanović, 2018 | Serious risk | Low risk | Low risk | Low risk | Low risk | Moderate risk | Low risk | Serious risk |
| Xu, 2020 | Moderate risk | Low risk | Low risk | Low risk | Low risk | Moderate risk | Low risk | Moderate risk |

**RoB-2 Tool Assessment**

| First author & year published | **Risk of bias arising from the randomization process** | Risk of bias due to deviations from the intended interventions (*effect of assignment to intervention*) | Risk of bias due to deviations from the intended interventions (*effect of adhering to intervention*) | Missing outcome data | Risk of bias in measurement of the outcome | Risk of bias in selection of the reported result | Overall risk of bias |
| --- | --- | --- | --- | --- | --- | --- | --- |
| Bahrami, 2016 | Some concerns | Low risk | High risk | Low risk | Low risk | Low risk | High risk |
| Hsu, 2021 | High risk | Low risk | Low risk | Low risk | High risk | Low risk | High risk |
| Mohanty, 2019 | High risk | Some concerns | N/A | Low risk | Some concerns | Low risk | High risk |
| Perić, 2022 | Low risk | Low risk | Low risk | Low risk | High risk | Low risk | High risk |
| Phung, 2019 | Some concerns | High risk | Low risk | Low risk | Low risk | Low risk | High risk |
