# Supplementary material for: Effects of group-based physical activity programs on children, adolescents, and young adults with disabilities: A systematic review
Source: PLoS One. 2025 May 23;20(5):e0323707. doi: 10.1371/journal.pone.0323707 (PMC12101651; doi:10.1371/journal.pone.0323707)
Supplement: S7 Table — (DOCX) [file pone.0323707.s010.docx]

**S7 Table. Summary of Extracted Data**

All studies were confirmed for eligibility by D.J and V.P-L as of date: 2023-08-29. Multiple additional searches were conducted in the respective databases and through handsearching to update the review between 2024 and January 15, 2025. No new articles meeting the inclusion criteria were identified.

Missing data were clearly identified in each supplementary file. When essential information (e.g., study characteristics or outcome data) was insufficient to interpret the results in line with the objectives of this review, the study was excluded from the systematic review.

Each of the included studies was assessed for the following information:

| **Description** | **Information displayed** | **Extractors** | **Extraction Date** |
| --- | --- | --- | --- |
| General description of the programs (S2 Table) | Participants details (sex, age, group details, disability type), study design and study quality. | Jason D’Amours & Pierre-Luc Veillette | Between 2023-02-11 to 2023-08-29 |
| Programs settings (S3 Table) | Program description  (program’s name, length, frequency, intensity, physical activity, trainers, training, group setting, additional details, fidelity of implementation) and program recommendations. | Jason D’Amours | Between 2023-08-29 to 2023-09-12 |
| Adaptation details (S4 Table) | Any information regarding how the intervention was adapted to accommodate participants’ respective disabilities was extracted, when available. | Jason D’Amours | Between 2023-08-29 to 2023-09-12 |
| Programs outcomes (S5 Table) | Variables measured, assessment tools (e.g., standardized scales), and main outcomes. | Jason D’Amours | 2025-01-18 |
| Risk of Biais (S6 Table) | Completed risk of bias and quality/certainty assessments for each study or outcome. | Jason D’Amours & Pierre-Luc Veillette | Between 2023-08-29 to 2023-12-23 |
